# Genome-wide mapping of histone H3K9me2 in acute myeloid leukemia reveals large chromosomal domains associated with massive gene silencing and sites of genome instability

**DOI:** 10.1371/journal.pone.0173723

**Published:** 2017-03-16

**Authors:** Anna C. Salzberg, Abigail Harris-Becker, Evgenya Y. Popova, Nikki Keasey, Thomas P. Loughran, David F. Claxton, Sergei A. Grigoryev

**Affiliations:** 1 Division of Biostatistics and Bioinformatics and Penn State Institute for Personalized Medicine, Hershey, Pennsylvania, United States of America; 2 Penn State College of Medicine, Dept. Biochemistry & Molecular Biology, H171, Hershey, Pennsylvania, United States of America; 3 Penn State College of Medicine, Department of Neural and Behavioral Sciences, Hershey, Pennsylvania, United States of America; 4 Penn State Cancer Institute; Milton S. Hershey Medical Center, 500 University Drive, Hershey, Pennsylvania, United States of America; 5 University of Virginia Cancer Center, Charlottesville, Virginia, United States of America; Southern Illinois University School of Medicine, UNITED STATES

## Abstract

A facultative heterochromatin mark, histone H3 lysine 9 dimethylation (H3K9me2), which is mediated by histone methyltransferases G9a/GLP (EHMT2/1), undergoes dramatic rearrangements during myeloid cell differentiation as observed by chromatin imaging. To determine whether these structural transitions also involve genomic repositioning of H3K9me2, we used ChIP-sequencing to map genome-wide topography of H3K9me2 in normal human granulocytes, normal CD34+ hematopoietic progenitors, primary myeloblasts from acute myeloid leukemia (AML) patients, and a model leukemia cell line K562. We observe that H3K9me2 naturally repositions from the previously designated “repressed” chromatin state in hematopoietic progenitors to predominant association with heterochromatin regions in granulocytes. In contrast, AML cells accumulate H3K9me2 on previously undefined large (> 100 Kb) genomic blocks that are enriched with AML-specific single nucleotide variants, sites of chromosomal translocations, and genes downregulated in AML. Specifically, the AML-specific H3K9me2 blocks are enriched with genes regulated by the proto-oncogene *ERG* that promotes stem cell characteristics. The AML-enriched H3K9me2 blocks (in contrast to the heterochromatin-associated H3K9me2 blocks enriched in granulocytes) are reduced by pharmacological inhibition of the histone methyltransferase G9a/GLP in K562 cells concomitantly with transcriptional activation of *ERG* and *ETS1* oncogenes. Our data suggest that G9a/GLP mediate formation of transient H3K9me2 blocks that are preserved in AML myeloblasts and may lead to an increased rate of AML-specific mutagenesis and chromosomal translocations.

## Introduction

In eukaryotic chromatin, the DNA is repeatedly coiled around octamers of small basic proteins, histones, to form nucleosomes [1]. Chromatin structures and posttranslational modifications of histones are key factors for understanding the expression of the underlying genes [2]. Heritable alterations in gene expression patterns resulting from changes in post-translational modifications of histones, DNA methylation, and the repertoire of transcriptional regulatory factors are referred to as epigenetic changes [3]. Epigenetic changes that orchestrate the transition of cells from an immature, proliferating state to mature, differentiated tissue are critical events in development, and genes regulating these processes are frequent targets of DNA mutations and epigenetic defects that may lead to cancer [4–8]. One example is acute myeloid leukemia (AML), a highly malignant hematopoietic disorder that comprises about 30% of total cases of leukemia and has the lowest survival rate among the four major types of leukemia [9]. AML manifests an interrupted process of hematopoiesis within bone marrow, resulting in overpopulation of the peripheral blood by immature myeloid cells, whose differentiation is blocked or stalled at different early stages. While normal hematopoiesis is regulated by a combination of extracellular signaling and intrinsic epigenetic changes [10], the fact that among about a the genes most frequently mutated in AML [11], the majority are known as epigenetic chromatin regulators indicates that chromatin epigenetics is the primary target of mutations causing AML as well as other hematological malignancies [12].

While DNA methylation and Polycomb repressive complexes are among the most studied epigenetic factors related to human leukemia [10, 12], in this work, we focused our studies on the role of a functionally related histone modification, histone H3 lysine 9 dimethylation (H3K9me2), in the processes associated with normal myeloid cell differentiation and its interruption leading to AML. This histone modification is associated with gene repression [13] and, in contrast to the association of a closely related repressive mark, histone H3K9 tri-methylation (H3K9me3), with constitutive pericentromeric heterochromatin, H3K9me2 marks repressed genes widely distributed in the euchromatin [14] and with the dynamic nuclear lamina associated domains (LADs) at the nuclear periphery [15]. By genomic analysis, H3K9me2 is significantly increased during normal cell differentiation [16, 17] to form Large Organized Chromatin K9 domains (LOCKs) that are notably altered in cancer cells [5, 18]. Visualized by immunofluorescence imaging, H3K9me2 is widely spread over the nuclear chromatin in actively proliferating cells, but it becomes spatially segregated from active euchromatin and structurally modified during terminal cell differentiation so that its antigenic exposure is blocked [19–21]. The above findings are consistent with H3K9me2 being a major mark of facultative chromatin that becomes condensed and repressed in a developmentally-regulated manner [21, 22].

Most of H3K9me2 modification in mammalian cells is mediated by the euchromatin histone methyltransferase (HMTase) G9a/GLP (EHMT2/EHMT1), which is essential for mouse embryonic development and embryonic stem cell differentiation [23], lineage commitment in human hematopoietic progenitor cells [17], and restricting reprogramming to pluripotent stem cells [4]. Loss of G9a eliminates H3K9me2 in euchromatin almost completely but does not affect H3K9 trimethylation in heterochromatin [14, 23].

G9a/GLP and histone H3K9me2 methylation are involved in repression of multiple genes associated with AML [24–26] but the functional role(s) of H3K9me2 in myeloid leukemogenesis is not clear. On one hand, H3K9me2 spreads during hematopoietic progenitor cell commitment and inhibition of G9a/GLP reduces their differentiation and promotes stem cell characteristics [17]. Accordingly, a recent study showed that JMJD1C, a demethylase reducing H3K9me2 levels, promotes survival of human AML cell lines [27]. On the other hand, pharmacological and genetic targeting of the HMTase G9a/GLP was shown to be efficient in slowing down AML cell proliferation in a mouse model and human AML cell lines [26, 28] thus making these HMTases potential targets for epigenetic therapy of AML. It thus appears that H3K9me2 may have both leukemia-promoting and leukemia-restricting role depending on genomic context and specific activity of histone-modifying enzymes.

Here we asked whether the genome-wide positioning of H3K9me2 is altered in the human AML cells as opposed to the normal process of H3K9me2 spreading during myeloid differentiation, do these changes include any specific genomic regions showing gains or loss of H3K9me2 in all or certain types of AML, are these changes associated with coordinated transitions in gene expression and/or genomic instability, and whether these changes, especially those specific for AML, could be reversed by pharmacological targeting of G9a/GLP.

To answer these questions, we developed a new approach to genome-wide analysis of large H3K9me2 blocks (**Fig A in S1 File**) and applied it to human myeloid cells including mature granulocytes, CD34+ progenitor cells, cultured K562 myeloid cells, and 10 samples of primary cells from the peripheral blood of AML patients. We identified AML-specific blocks of H3K9me2 (termed differential Large Organized Chromatin K9 domains or dLOCKs) coordinated with large-scale transcriptional changes in AML. We also observed wide-scale reduction in H3K9me2 levels associated with transcriptional changes following pharmacological inhibition of G9a/GLP in the leukemia model cell line K562. Strikingly, the new AML-specific H3K9me2 blocks contained a number of down-regulated proto-oncogenes, in particular genes transcriptionally regulated by ERG, a factor promoting adult hematopoietic stem cell self-renewal and expansion [29, 30], suggesting that the spreading of H3K9me2 blocks in AML may restrict stem cell characteristics by inactivating ERG and its downstream targets.

In addition, the AML-enriched H3K9me2 blocks exhibited an increased occurrence of AML-specific single nucleotide variations (SNV) and sites of chromosomal translocations known to cause AML. Provided that myeloid differentiation is essential for initiation of AML [31], we propose that the AML-specific H3K9me2 blocks may contribute to promoting AML by increasing chromosomal instability and mutagenesis at the chromosomal regions enriched with repressed protooncogenes. We expect that the observed epigenomic transitions may serve for AML prognosis as well as targets for its epigenetic therapy and prevention of chromosomal instability.

## Materials and methods

### Fractionation and isolation of human blood cells and cell nuclei

Normal polymorphonuclear granulocytes (predominantly neutrophils) were isolated from discarded fresh white blood cells (buffy coats) from unidentified healthy donors collected at Hershey Medical Center Blood Bank (IRB protocol # HY03-136EP-A) using standard OptiPrep density centrifugation [32] and resuspended in PBS (Phosphate Buffered Saline) buffer. The AML blood samples to be used in this study were collected from patients with written informed consent at time of sample collection under guidelines and procedures approved by institutional review board of Milton Hershey Medical Center (IRB protocol 2000–186; STUDY00002518). D. F. Claxton, M.D., the attending physician, was responsible for de-identifying the samples collected from the patients. AML mononuclear cells were collected from peripheral blood of 10 patients diagnosed with AML with and without monocytic features (FAB types 1, 2, 4, and 5) at the time of diagnosis prior to treatment, isolated by Ficoll-Pacque density gradient centrifugation [33], cryopreserved in RPMI media with 10% dimethylsulfoxide and 5% human serum albumin (American Red Cross, Washington, DC, USA) and stored in liquid nitrogen. For clinical classification, the samples were subjected to flow cytometry and percent of surface expression of various antigens is given in **S1 Table**. Cryopreserved bone marrow CD34+ cells from normal donors were obtained from Allcells (Alameda, CA). Bone marrow CD34+ cells represent a combination of stem and progenitor cells and can be easily isolated at high cell numbers without cytokine mobilization [34]. For a scheme relating the types of AML to the process of human hematopoiesis, see **Fig A in S1 File.**

Human K562 cells (ATCC CCL-243) were grown in RPMI 1640 GlutaMAX medium (Invitrogen) supplemented with 10% FBS (HyClone, Logan, UT) and 1% penicillin-streptomycin (Invitrogen), in 5% CO_2_ at 37^°^C. K562 cells were grown for not more than 10 passages after acquiring from ATCC. K562 cells were treated with 0–1 μM UNC0638 (a G9a/GLP HMTase inhibitor; Sigma U4885), with 0.01% (vol/vol) DMSO, for 5 days; medium was replaced on day 3 with fresh growth medium containing UNC0638 or DMSO vehicle only. Cells were incubated with UNC0638 at the concentration indicated, when not specified the K562 cells were treated with 1 μM UNC0638. Three biological replicates were performed, using independently cultured K562 cells. After treatment, K562 cells were washed 2 x PBS and then resuspended in PBS for fixation.

### Antibodies

For chromatin immunoprecipitation (ChIP) and western blotting analyses, we have used anti-H3K9me2 (ab1220), anti-H3K9me3 (ab8898), anti-H3 C-tail (ab8898) from Abcam; and Anti-H3K4me2 (07–030) from Upstate/Millipore. We have successfully tested these antibodies for immunofluorescence analysis, western blotting, and ChIP in previous works with myeloid [32] and other cells types [20, 35]. For quality assessment of those antibodies in ChIP applications, see [36].

### PAGE and western analysis

SDS-PAGE was carried out in 15% polyacrylamide and the proteins were transferred to Immobilon-P PVDF membranes (Millipore) in Tris-glycine buffer containing 10% methanol. Membranes were blocked in 3% nonfat dry milk in TBS buffer with 0.1% Tween 20, and incubated with primary anti-H3K9me2 (ab1220), anti-H3K9me3 (ab8898), and anti-H3 C-tail (ab8898) from Abcam as previously described [32]. Semi-quantitative analysis of relative protein levels was performed using ECL Prime (Amersham, GE Healthcare) and photographic film, which was digitized and the intensity of protein bands was quantitated using ImageJ [37].

### Chromatin immunoprecipitation

For formaldehyde fixation, cultured cells or freshly thawed cells were suspended in PBS at ~ 5x10^7^ cell/ml at room temp., mixed with 1/10 of the final volume of 10% formaldehyde in PBS freshly prepared from 37% stock (Fisher, ACS reagent F79-500). Cells were fixed with 1% formaldehyde for 10–15 min at room temp. Fixation was stopped by adding of 125 mM glycine. Cells were spun down at +4^°^C and 4000 g for 5 min. and washed 3 times cold PBS. The fixed cells were counted to estimate the yield and stored overnight at +4^°^C.

Nuclei from formaldehyde-fixed cells were isolated as described [38]. The purified nuclei were resuspended in micrococcal nuclease digestion buffer (50 mM Tris-HCl, pH = 7.6; 3 mM CaCl_2_) at ~1 mg/ml DNA. The nuclear suspensions (1 ml) were digested with 60 units of Micrococcal nuclease (Roche) for 40 min. at + 37^°^C to ~200 bp nucleosome sizes and digestion was stopped by adding 3 mM EDTA. After digestion, the supernatant (S1) was collected and discarded and the nuclear pellet (P) was resuspended in SDS-containing Lysis buffer (1% SDS; 10mM EDTA; 50 mM Tris HCl pH 8.0) with light sonication (Fisher model 100, 2 x 10 sec. at setting 2) to enhance solubility rather than to shear DNA. Chromatin immunoprecipitation (ChIP) was conducted essentially as described [39]. The final DNA was dissolved in 50–100 μl DNase-free water. DNA concentration was measured using Qubit fluorometer (Invitrogen).

For each sample, we conducted two parallel immunoprecipitation reactions with a) no antibody added; b) similarly prepared IgG from a none-immunized animal (e.g. rabbit IgG #2729, Cell Signaling). A typical yield from immunoprecipitation with antibodies against H3K9me2 (Abcam, ChIP Grade, ab1220) was more than 400 ng for 40 μg of the input (ChIP/Input ratio >1%). Nonspecific binding in the control samples was < 0.02%.

### Library preparation and Next-generation sequencing

For SOLiD sequencing, the DNA samples (IP ChIP and IP input, ~ 100 ng each) were submitted for preparing fragment libraries and their amplifications using Applied Biosystems reagents and deep sequencing (~20x10^6^ 70 bp DNA sequence reads per sample) using the SOLiD sequencer at the Penn State Genomic Core Facility (University Park). For next-generation Illumina sequencing library preparations, we used NEBNext ChIP-Seq single-end library preparation reagent set for Illumina (cat #E6200) and NEBNext Multiplex Oligos for Illumina Index Primers Set 1 (cat #E7335). For one library, we took 10–30 ng of chromatin-immunoprecipitated or input DNA. End repair of ChIP DNA, dA-Tailing, Adaptor ligation, and cleaning of DNA by AMPure XP magnetic beads (Agencourt part # A63880), were conducted according to the NEBNext ChIP-Seq library preparation manual with exception that after adaptor ligation and AMPure cleaning (step 4), the material was eluted in 20 μl of 0.1 x TE buffer for agarose gel size selection. DNA electrophoresis was run in 2% agarose gels with SyBr Gold gel stain (Invitrogen, S11494) using TAE buffer and 100 bp DNA ladder (NEB N3231S) as size standard. We excised a band from the gel ranging in size from 175–225 bp (150 bp nucleosome plus 50 bp adaptors) using the DNA ladder as a guide. We purified the DNA using Qiagen QIAquick gel extraction kit (cat# 28704) and eluted in 50 μl of sterile water. We took 23 μl of eluted size-selected DNA for PCR enrichment using the universal primer and one of 12 index primers (from NEB cat #E7335) as described in the NEB Next ChIP-Seq library preparation manual. We used 15–17 cycles of PCR amplification depending on the amount of starting material. After PCR, we cleaned the PCR-amplified library DNA by AMPure XP magnetic beads, redissolved in 20 μl of 0.1 x TE buffer and measured the DNA concentration using Qubit fluorometer (dsDNA HS assay). Typical yield was 40–100 ng/μl. For quality controls, we assessed the DNA size distribution on a Bioanalyzer^®^ (Agilent, High Sensitivity DNA Assay). The DNA libraries were sent for deep sequencing using the Illumina Hi-Seq 2500 sequencer at Penn State Genomic Core Facility (University Park). We used rapid-run mode with 2 lanes, 4 samples per lane and 100 nucleotides per read. For each sample Illumina sequencing provided 32–40 million of 100 bp reads (see **S2 Table** for SOLiD and Illumina-sequenced samples).

### Mapping to the human genome and H3K9me2 domain calling

Sequence reads were mapped to the hg19 assembly of the human genome using bowtie 0.12.9 with default parameters (see GEO database acc.# GSE71809 for the raw and processed sequence reads files associated with this study). Domains were called with the RSEG software, version 0.4.8 RC, using the provided deadzone files k41-hg19 [40]. The size domains were calculated for the RSEG predicted values of 10 kb, 20 kb and 50 kb and appeared to be almost identical (see **Fig B in S1 File**). The RSEG domains calculated with predicted 10 kb values were used in this study. Other parameter used was: “keep duplicates”. The chromosome X and Y mappings were duplicated for the male samples prior to calling RSEG. Sequence reads mapped to multiple genomic locations were excluded from subsequent analysis. The RSEG enriched domains were mapped into windows of sizes 10 kb, 50 kb, 100 kb, 500 kb and 1Mb, with each domain contributing to a window’s score its average read count for each of its nucleotides falling within the window. Window scores for each dataset were then normalized by the average score of all its windows and technical replicates were then averaged. Supplementary files showing enriched RSEG domains and their boundaries are included in the GEO database (acc.# GSE71809) and contain “rsegout.bed.gz” in their filename extensions.

### Hierarchical cluster analysis

Cluster analysis of H3K9me2 sequence read distributions genome-wide and on specific chromosomal loci was done with open source Gene Cluster 3.0 software [41] with hierarchical clustering by complete linkage and visualized either with TreeView version 1.6 (2002) or with Java Treeview 1.1.6r2 (2011).

### Correlations and fold enrichment analyses with genomic data from databases

The following human ENCODE [42] datasets were extracted using the University of California, Santa Cruz (UCSC) browser (http://genome.ucsc.edu) table tool: 1–16: chromatin state segmentation by HMM from ENCODE/Broad Institute [43]; 17, 18: European Institute of Oncology/J.C.Venter Institute CD34 DNase I EIO/JCVI CD34+/- Nuclease Accessible Sites, CD34+ mobilized UW DNase I HS hotspots and peaks [44]; 19–24: University of Washington DNaseI clusters, CD34+ mobilized DNaseI hotspots and peaks, and K562 DNaseI hypersensitive peaks [45]; 25: NKI LADs laminB1Lads [46]; 26–28: Transcription Factor Binding Sites for ZEB1, KAP1, and clustered TFBS from ENCODE/Analysis ChIP-seq (40); 29: Target Scan miRNA regulatory sites [47]; 30, 31: Hudson Alpha Institute for Biotechnology K562 Transcription Factor Binding Sites for USF1 [48], 32–39: Broad Institute K562 CTCF, POL2, histone H2AZ, histone H3K27me3, histone H3K4me2, histone H3K9me1, histone H3K9ac [43]; 40–42: Hudson Alpha Institute for Biotechnology K562 CpG methylation by Methyl 450 bead arrays and Reduced Representation Bisulfite Sequencing (RRBS) sites, [49]; 43: CpG islands [50]; 44: vista enhancers (functionally active enhancers) [51]. Additional public datasets used were, 45: H3K9me2 in T lymphocytes [52], 46: cancer SNVs [53]; 47: AML-specific SNVs [11]; 48: Long-range epigenetic silencing in cancer cells, LRES [54]. We also calculated: 49—density of all annotated genes in hg19 per 10 kb windows and 50—density of chromosomal translocation and inversions affecting specific genes per 10 kb windows. The chromosomal translocation density (see **S3 Table**) was derived from the list of all AML-specific chromosomal translocations and inversions in the Atlas of Genetics and Cytogenetics in Oncology and Haematology (http://atlasgeneticsoncology.org/index.html). For correlation analysis, the RSEG k41-hg19 deadzones [40] were subtracted from each of our experimental, public and computed datasets and then mapped into windows of sizes 10 kb, 50 kb, 100K, 500 kb and 1Mb for chromosomes 1–22 (see **S4 Table**). Pairwise Pearson correlations were calculated using R 2.15.1.

### Identification of conserved and variable H3K9me2 domains

Conserved high (“Red”) and conserved low (“Green”) H3K9me2 domains were determined by calculating the standard deviation of the 10 kb window scores of the H3K9me2 granulocyte, CD34+, and AML samples. Only the male samples were used to determine the conserved domains over the Y chromosome. A threshold of 1 standard deviation was used to classify a sample’s window as ‘high’ (score > stdev) or ‘low’ (score ≤ stdev). An overall window was classified as conserved low (“Green”) H3K9me2 domains when only 2 or less samples were ‘high’ and at most one was ‘high’ from each of the granulocyte, AML, and CD34+ categories. Analogously, a window was classified as conserved high (“Red”) when only 2 or less samples were ‘low’ with at most one ‘low’ from each category. All remaining windows were classified as variable H3K9me2 domains (“Yellow”).

### Identification of differential blocks of H3K9me2 (dLOCKs)

To set bases for dLOCK calculations, the 10 kb window RSEG entries were grouped into the following categories: a) granulocytes, b) CD34+ c) K562 control, d) K562-UNC0638 treated, e) AML cluster A, f) AML cluster B. For each group we counted a continuous stretch of 10 kb windows as a LOCK base if at least 50% of 10 kb windows in each row contained positive values. For each of the continuous LOCK bases, for category “c1” above (where c1 is one of a-f), a dLOCK c2>c1 10 kb window score was computed as the sum of the negative log ratio (Sum Log Ratio) of the 10 kb window score of a sample in the first listed category to that of a sample in the second for every possible first and second category sample pair. This result was then normalized by the number of pairwise terms in the sum. The X and Y chromosome dLOCK 10 kb sums only included terms of sample pairs having the same gender.

Contiguous blocks of 10 kb windows were determined to be a positive dLOCK of size spanning those windows if the total score, defined to be the sum of the corresponding dLOCK 10 kb window scores, was positive, and a negative dLOCK for a negative total score. The threshold of significant positive dLOCKs was set to the 90 percentile, and that of significant negative dLOCKs to the 10 percentile. The latter calculation was performed using the ‘quantile’ function in R, with alpha = 0.90 and 0.10 respectively excluding Y chromosome LOCKs. We thus calculated ten categories of differential LOCKs (dLOCKs): 1. CD34+ > granulocytes; 2. granulocytes > CD34+; 3. CD34+ > AML cluster A; 4. AML cluster A > CD34+; 5. granulocytes > AML cluster A; 6. AML cluster A > granulocytes; 7. CD34+ > AML cluster B; 8. AML cluster B > CD34+; 9. K562 UNC0638 > K562 control; 10. K562 control > K562 UNC0638; each category including ALL dLOCKs, and dLOCKs with alpha <0.10, >0.90, <0.05, >0.05, <0.01, >0.99 (**S5 Table**). For 10 categories of dLOCKs as well as for the 3 genomic zones as well as for each of the Red, Green, and Yellow genomic fractions, we calculated the enrichments of the genomic features from UCSC/ENCODE databases (**S6 Table**).

### Pathway, gene ontology and upstream regulator analyses

For each dLOCK set described above, the genes of the top 1%, 5% and 10% scoring dLOCKs (alpha <0.01 and >0.99, <0.05 and >0.95, <0.10 and >0.90) were extracted (see **Table A in S7 Table**). Ingenuity® Pathways (IPA build version 212183) was used to identify upstream regulators and significantly enriched canonical pathways, networks, diseases and disorders, molecular and cellular functions, physiological system development and functions, and toxicity lists. Ingenuity Pathway was used with the following parameters: reference set to Ingenuity Knowledge Base Genes Only, relationships to include set to direct and indirect, includes endogenous chemicals, and filter set to only consider molecules or relationships for the human species and where the confidence is set to experimentally observed.

### mRNA expression analysis

Total RNA was isolated from cryopreserved AML cells and human bone marrow CD34+ cells from normal donors (Allcells, Alameda, CA) using Trizol® reagent (Invitrogen, 15596–026) with 1 ml of Trizol reagent per 5–10 x 106 cells as described in the vendor’s manual. Isolated RNA was quantified using the Bioanalyzer RNA 6000 Nano Assay kit (Agilent Technologies, Santa Clara, CA). Four AML type A and two AML type B samples randomly chosen from initial 10 AML samples and 6 normal donor samples were taken for this analysis. First strand cDNA was produced from 1.0 μg of total RNA using the standard protocol from the High Capacity cDNA Reverse Transcriptase kit (Life Technologies, Grand Island, NY). cDNA concentrations were quantified by absorbance using a NanoDrop ND-1000 Spectrophotometer (Fisher Scientific, Pittsburgh, PA). cDNA expression rates in AML were assayed using Illumina HumanHT-12 array containing hybridization probes with 33,732 distinct chromosomal positions and using normal bone marrow CD34+ cells as controls (see **Table D in S7 Table**).

### Genomic qPCR, RT-qPCR and PCR primers

All quantitative real-time PCR (qPCR) was performed using FastStart Universal SYBR Green Master (Roche) on a Roche LightCycler 480 Instrument following the manufacturer’s instructions.

For ChIP-qPCR assays, reactions were performed in triplicate, with two technical replicates for each sample. The fold enrichment of ChIP over input was calculated as 2 to the power of the quantification cycle (Cq) difference between input chromatin and ChIP samples. Genomic qPCR primers are listed in **S8 Table**.

For RT-qPCR, total RNA was extracted using Trizol (Invitrogen) and Direct-zol RNA MiniPrep kit (Zymo Research), and cDNA was synthesized using SuperScript II First-Strand Synthesis System and oligo(dT) (Invitrogen). Reactions for each target were performed in triplicate, with two technical replicates for each sample. Melt curve analysis was performed at the end of PCR to confirm the presence of a single, specific product. RT-qPCR primers are listed in **S8 Table**. The (Δ(ΔCq)) method was used to calculate target mRNA levels relative to two housekeeping genes, GAPDH and TBP, with similar results; data relative to GAPDH is shown. Fold change in gene expression was calculated by comparing DMSO control- and UNC0638-treated K562 cells. Student’s T-Test was used to evaluate the statistical significance of RT-qPCR gene expression analysis in two independent biological replicates.

## Results

### Myeloid cells contain long chromosomal blocks of H3K9me2 that have low gene density and are sustained during myeloid differentiation

To determine genome-wide rearrangements of H3K9me2, we conducted ChIP with anti-H3K9me2 and several control antibodies with normal human granulocytes, normal bone marrow CD34+ hematopoietic progenitors, and K562 human myeloid leukemia cell line (see experimental flowchart in **Fig A in S1 File**). K562 is a blastic myeloid leukemia cell line that has been extensively characterized as a tier I model cell of the human ENCODE project [43] thus facilitating its use in genome-wide data analysis.

**Fig 1:**
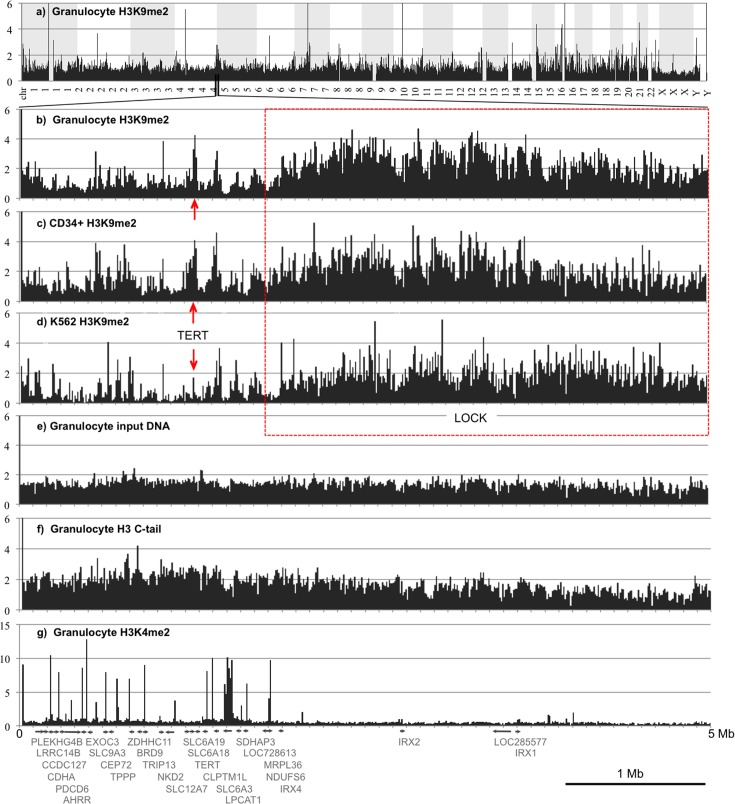
Human myeloid cells contain conserved blocks of H3K9me2 at gene-depleted chromosomal regions. Distribution of DNA sequence reads mapped to the human genome at 1Mb resolution (a) and at 10 kb resolution (showing a region of chromosome 5 between 0 and 5 Mb (b-g). DNA sequence reads were derived from H3K9me2 ChIP-seq of granulocytes sample 15 (a, b), bone marrow CD34+ cells sample 20 (c), K562 cells sample 3 (d); granulocyte input DNA sample 23 (e), granulocyte ChIP-seq of H3 C-tail sample 16 (f), and granulocyte ChIP-seq of H3K4me2 sample 28 (g). The reads were grouped by 10 kb windows and normalized to genome average over the total human genome. Note enrichment of H3K9me2 over a large chromosomal domain, or LOCK [18] indicated by red dashed line box and variable levels of H3K9me2 over the *TERT* gene forming a peak in the granulocyte and CD34+ cells but not in K562 cells indicated by vertical red arrows. Y-axes represent fold enrichment of sequence reads vs. genome average.

Most ChIP-sequencing and mapping experiments were independently repeated on two sequencing platforms (SOLiD and Illumina) each resulting in about 20–30 mln. reads per sample (**S2 Table**) to ensure that the results of data analysis are independent of the next generation sequencing techniques. The sequence reads (omitting duplicated sequences) were aligned to the human genome hg19 (see GEO database acc.# GSE71809 for the sequence reads files associated with this study). At the chromosomal scale, the H3K9me2 ChIP reads were distributed evenly, with exception of the haploid X and Y chromosomes in males (**Fig 1A**).

**Fig 2:**
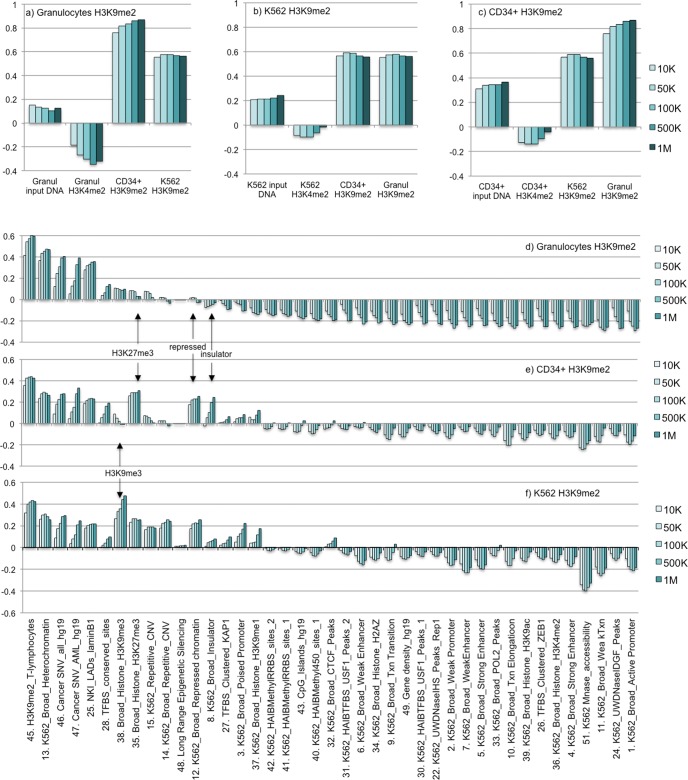
Genome-wide correlation analysis of H3K9me2 domains in myeloid cells. **a-c**): graphs showing Pearson correlation values for normal granulocytes (a), K562 (b), and CD34+ H3K9me2 (c) ChIP HMM domains vs: input DNA, H3K4me2 ChIP HMM domains, and H3K9me2 ChIP HMM domains from the two other samples as indicted. The five columns in each sample show correlation values for 10K, 50K, 100K, 500K, and 1M windows (left to right). **d-f**): graphs show Pearson correlations between granulocyte (d), CD34+ (e), and K562 (f) H3K9me2 HMM domains vs. indicated biodata from human genome-wide databases (for references see Materials and Methods and **S4 Table**).

At the higher resolution, the H3K9me2 ChIP-seq reveals profound variations in the distribution of H3K9me2. A map of a chromosome 5 region in granulocytes at 10 kb resolution (**Fig 1B**) shows an example of typical genome-wide distribution of H3K9me2 with low levels of this mark associated with a region of high gene density and an extended block of high level of H3K9me2 associated with a gene desert (underlined by a red line). Similar Large Organized Chromatin H3K9me2 domains (LOCKs) were described previously for human placenta cells [18] using ChIP-on-chip technology for selected regions of the human genome. The domain-wide organization of H3K9me2 is in sharp contrast to the even distribution of the input DNA (**Fig 1E)** and ChIP-seq for total histone H3 using antibodies against its unmodified C-tail (**Fig 1F**), as well as ChIP-seq showing sharp peaks of an active chromatin mark H3K4me2 (**Fig 1G**). The input DNA and the H3 C-tail levels appear to be somewhat lower at the regions with high H3K9me2 consistent with the recent observation that micrococcal nuclease accessibility and release of mononucleosomes negatively correlates with two other epigenetic markers of repressed chromatin, H3K27me3 and H3K9me3 [55] and our observation of an even stronger negative correlation of H3K9me2 with MNase sensitivity in K562 cells (see **Fig 2B** below). Furthermore, the ChIP-seq data normalized to input DNA and histone H3 C-tail also show the same constitutive LOCK in both cell types (**Fig C in S1 File**) thus attesting for unique and specific genome-wide distribution of the histone H3K9me2 mark in myeloid cells.

The positions of the large H3K9me2 blocks were generally conserved over the chromosomes of granulocytes, CD34+ progenitors, and K562 cells (cf. **Fig 1B–1D**) and apparently similar to LOCKs described in human placenta [18]. However, we were intrigued by observing some gene-containing chromosomal domains (for example a domain including the *TERT* gene repressed during granulocyte differentiation and reactivated in many types of cancer cells [56], which showed marked changes between normal myeloid cells and K562 (**Fig 1B–1D, and Fig C in S1 File**; vertical red arrows). Therefore, we decided to characterize the extent and levels of H3K9me2 domains in the normal and myeloid leukemia cells in relation to other genome-wide biological data available from databases.

### H3K9me2 domains in myeloid cells are enriched with lamina-associated domains (LADs) and cancer-related SNVs

To determine the genomic positions and boundaries of H3K9me2 domains and eliminate possible artifacts and repeats known as “deadzones” we used RSEG, an unbiased procedure for selecting domain boundaries based on hidden Markov model (HMM) analysis of genome-wide ChIP-seq read distribution [40]. First, we calculated RSEG domains for H3K9me2 distribution in K562 cells and analyzed co-localization between the boundaries of H3K9me2 domains with other functional genomic features from ENCODE/Broad Institute data [43]. We observed that the sharp transitions in the occurrence of transcription regulation sites (strong promoters and enhancers, and especially the peaks of CTCF, a protein known to demarcate the chromatin domain boundaries [57]) coincided with the boundaries of H3K9me2 domains showing a strong exclusion of these regulatory elements from the H3K9me2 domains. The weaker enhancers and promoters showed much more shallow transitions and hence less exclusion. Notably, H3K4me2, a marker of active genes, had a strong drop coinciding with the H3K9me2 boundaries in a sharp contrast with peaks of the two repressive marks, H3K27me3 and H3K9me3 **(Fig D in S1 File**).

For genome-wide correlation analysis of H3K9me2 in K562, granulocytes, and CD34+ cells and comparison with our experimental datasets and available data from human genome databases (see refs. 41–55), the RSEG domains were grouped by 10 kb, 50 kb, 100 kb, 500 kb, and 1Mb windows and Pearson correlations were calculated for all experimental and database entries (**Fig 2 and S4 Table**). Independent analysis of our experimental datasets showed a negative correlation of H3K9me2 with H3K4me2 in the three cell types (**Fig 2A–2C**) consistent with the boundary analysis. Interestingly, the two types of normal myeloid cells, granulocytes and CD34+ progenitors, both showed a higher positive correlation between themselves than with a leukemia cell line K562 (**Fig 2A–2C**) prompting us a more extensive analysis of H3K9me2 in human AML cell (below).

Correlation analysis with multiple genomic datasets revealed several general features consistent with the expected function of H3K9me2 in establishing repressive and/or heterochromatic chromatin states: 1) strong positive correlation of H3K9me2 in K562, granulocytes and CD34+ cells with H3K9me2 distribution in human lymphoid T-cells [52] thus showing that this histone modification is generally conserved between myeloid and non-myeloid cells; 2) positive correlations of H3K9me2 with H3K9me3 and H3K27me3 (the two repressive marks analyzed by ENCODE [43] is consistent with synergism between these three main types of repressive epigenetic marks [58, 59]; and 3) negative correlations with a number of activatory epigenetic marks, promoters, enhancers, transcriptional elongation, RNA polymerase II, gene density, and accessibility to DNase I and micrococcal nuclease (**Fig 2D and 2E**) thus confirming a tight association of H3K9me2 with chromatin states previously described as “repressed chromatin” and “heterochromatin” [43] in myeloid cells.

As expected from previous studies in non-myeloid cells [18, 53, 60], for all three myeloid cell types we observed strong positive correlations between H3K9me2 domains and LADs, total cancer SNV, and AML-specific SNV (**Fig 2 and S4 Table**). Remarkably, we observed a striking contrast between the strong positive correlation of H3K9me2 with the previously defined “repressed” chromatin state [43] and H3K27me3 in K562 and CD34+ cells and the very weak correlations in granulocytes (arrows on **Fig 2**). In addition, correlations with the insulator elements known to separate functional chromosomal domains [61] were notably discriminatory between CD34+ and granulocytes. H3K9me2 was strongly correlated with a constitutive heterochromatin mark H3K9me3 in K562 but not in granulocytes and CD34+ cells. No other biomarkers showed such strong differences among the myeloid cells (**Fig 2 and S4 Table**).

We thus concluded that the H3K9me2 domains form a set of conserved large organized chromosomal blocks (LOCKs) whose association with gene deserts, low nuclease accessibility, and regions of increased occurrence of cancer-related SNV (reflecting their relative inaccessibility to DNA repair [62]), is universal. The genome-wide distribution of H3K9me2 is clearly distinct from the other repressive heterochromatin marks (H3K9me3 and H3K27me3), and changes dramatically between the normal myeloid cells and leukemic K562 suggesting that the H3K9me2 domains may substantially rearrange during normal myeloid differentiation as well as leukemogenesis.

### Histone H3K9me2 undergoes genome-wide rearrangement in AML cells

To look for possible alterations of H3K9me2 in human myeloid leukemia, we conducted ChIP-seq analysis of 10 different human AML samples, mostly cases with cytogenetically normal AML (**S1 Table**). For all AML H3K9me2 domains, we observed strongly positive (r > 0.5) genome-wide correlations between the individual AML cases as well as with the “normal” human samples–granulocytes and CD34+ progenitors (S4 **Table**). As controls, the SOLiD/Illumina platforms replicate correlations were in the range r>0.671 to 0.805 thus attesting for independence of the correlation analysis from the sequencing platforms.

**Fig 3:**
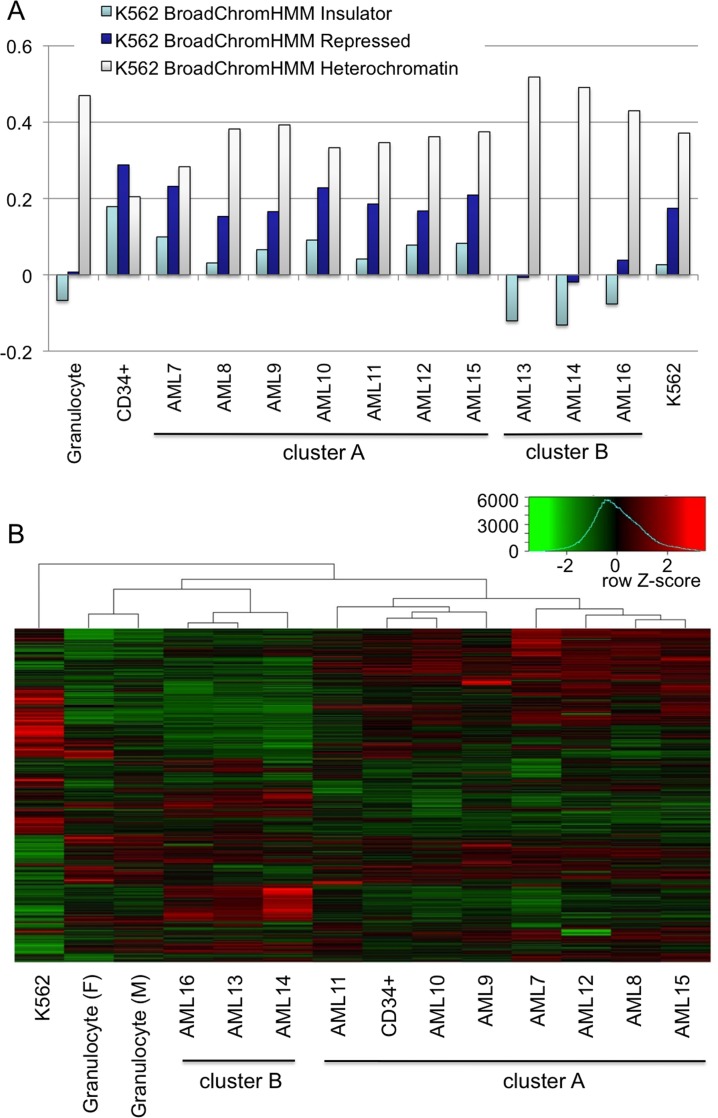
Analysis of H3K9me2 correlations in AML cells and identification of two epigenetically different types of AML. A: Graphs showing correlations between Granulocyte, CD34+ progenitors, 10 AML samples, and K562 H3K9me2 HMM domains vs. selected biodata with top discriminatory power (insulator, repressed chromatin, and heterochromatin). B: Hierarchical cluster analysis of genome-wide H3K9me2 distribution for 10 AML samples, CD34+, granulocytes, and K562 cells. The insert on top shows the color key and histogram for H3K9me2 enrichment/depletion score.

Comparison of H3K9me2 domains in AML with multiple genomic biodata showed a wide variation in H3K9me2 correlations with two principle chromatin states [43]: insulators and repressed chromatin (**Fig 3A** and **S4 Table**). Seven AML samples (AML7-12, 15) displayed a positive H3K9me2 correlation with repressed chromatin and insulators thus closely resembling CD34+ progenitors. In contrast, the other 3 AML samples (AML13, 14, 16) had negative or very weak correlation with insulators and repressed chromatin, more similar to granulocytes than to CD34+ and the other AML samples.

Furthermore, a non-supervised cluster analysis of the H3K9me2 HMM domains showed that the AML samples clearly segregate into two distinct clusters (**Fig 3B)**. One cluster designated here as AML cluster A (high level of correlation between H3K9me2 vs. repressed chromatin on **Fig 3A**) contained all 3 CD34-positive cases of AML with features of less differentiated cells. The other cluster, designated AML cluster B (strong correlation with heterochromatin) showed features of more differentiated AML M4 and M5 and was characterized by high monocytic cell counts (**S1 Table)**.

### Identification of large variable blocks of H3K9me2 (dLOCKs) associated with myeloid differentiation and leukemogenesis

**Fig 4:**
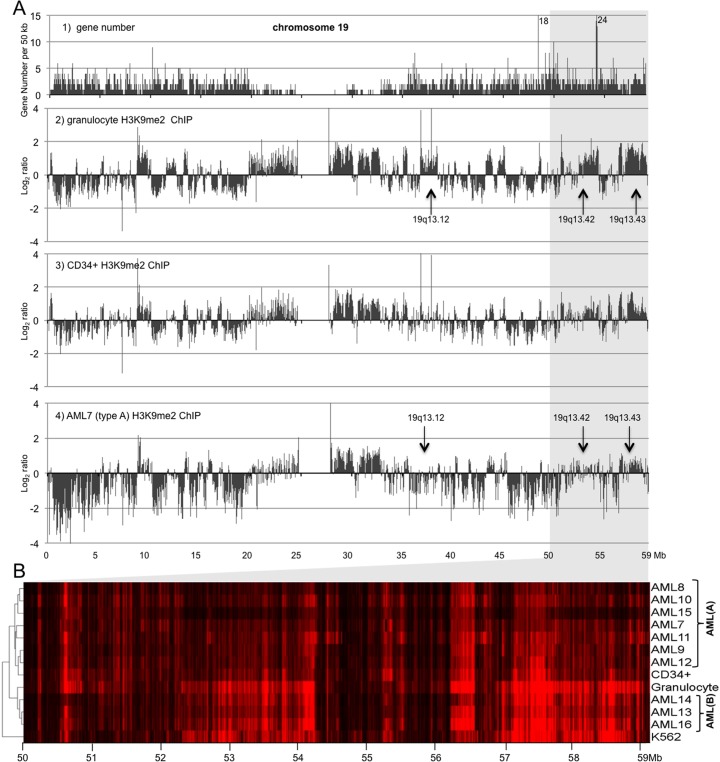
Mapping of H3K9me2 blocks shows consistent alterations associated with myeloid differentiation and AML. A: 50K resolution maps of chromosome 19 showing gene density (1) and H3K9me2 ChIP-sequence reads (Bowtie) of human granulocytes (2), normal CD34+ cells (3), and AML myeloblasts (4). Sequence reads were normalized to average and plotted in Log_2_ scale. Red arrows show difference in H3K9me2 levels at three loci at chromosome 19. B: Hierarchical cluster analysis of H3K9me2 distribution over the chromosome 19 (50–59.12 Mb) locus for 10 AML samples, CD34+, granulocytes, and K562 cells.

In addition to the changes in genome-wide correlations, mapping of H3K9me2 revealed prominent changes in large blocks of H3K9me2 often containing multiple genes. A striking example of such blocks on chromosome 19 showing the strongest H3K9me2 changes between normal and AML cells (**Fig 4A**) contains dozens of genes encoding zinc finger (ZNF) proteins. An unsupervised cluster analysis of the chromosome 19 (50–59 Mb) locus (**Fig 4B**) showed the same two distinct clusters as above (**Fig 3B**), with AML cluster A showing strongly reduced H3K9me2 and AML cluster B showing distributions much closer to that of K562 cells and granulocytes. A 10 kb map of H3K9me2 HMM domains confirmed the differences between dLOCKs of the AML cluster A and cluster B (cf. panels c-h, k vs. i, j, l on **Fig E in S1 File**) and also showed that the differences revealed by ChIP reflected actual histone H3K9me2 level variations and not the genomic copy number variations since the input DNA maps of normal and AML cells were almost identical (**Fig F in S1 File, panels 5–6).** In addition, western blotting analysis showed no significant difference between the AML samples in general H3K9me2 levels (**Fig G in S1 File, panel a**). Furthermore, parallel ChIP-qPCR experiments with gene regions from selected chromatin regions in granulocyte and four AML type A and two AML type B samples randomly chosen from initial 10 AML samples were consistent with the results of ChIP-seq analysis (**Fig G in S1 File, panels b-h**) though qPCR data exhibited stronger individual variations than ChIP-seq. Therefore, in subsequent work, we focused our analysis of ChIP-seq data derived from AML cluster A, which is better represented in the current dataset.

The observation of large variable H3K9me2 blocks prompted us to conduct genome-wide comparative analysis of large blocks of H3K9me2. Therefore, we calculated differential LOCKs (dLOCKs) of H3K9me2 for six comparison categories: two representing epigenetic changes during normal myeloid differentiation (CD34+ > granulocytes and granulocytes > CD34+) and four representing epigenetic changes between AML and normal cells (CD34+ > AML type A; AML type A > CD34+; granulocytes > AML type A; AML type A > granulocytes). The dLOCKs are ranked by their Sum Log Ratios (see materials and methods) reflecting the extent of the developmentally-regulated and/or AML-related changes in H3K9me2 levels (**Table 1).** The threshold of significant dLOCKs was set to the 95 and 5 percentile using the ‘quantile’ function in R, with alpha = 0.95 and 0.05 respectively, excluding Y chromosome LOCKs. The list of all H3K9me2 dLOCKs with changes in Sum Log Ratio (alpha < 0.10 and alpha >0.90) is shown in **S5 Table.**

**Table 1:** Main characteristics of transient H3K9me2 blocks (dLOCKs) with strongest variations among myeloid cells.

dLOCK category	alpha	% of the genome	average dLOCK size, bp	number of genes	number of dLOCKs	gene density/ genome average	enriched genomic features (alpha > 0.99; alpha <0.01)	fold enrichment
**CD34+ >Granulocyte**								
							35. Histone H3K27me3	2.6
	>0.99	3.21%	487549	724	204	1.1	47. cancer SNV AML	2.46
	>0.95	9.48%	287959	2742	1019	1.04	3. Poised Promoter	2.17
	>0.90	13.97%	212223	4760	2038	1.01	37. histone H3K9me1	1.72
							8. Insulator	1.59
**Granulocyte > CD34+**								
							15. Repetitive CNV	1.76
	< 0.01	5.54%	695121	1063	248	0.76	47. cancer SNV AML	1.72
	< 0.05	15.17%	381246	2523	1236	0.74	38. histone H3K9me3 Peaks	1.7
	< 0.10	20.96%	263351	3568	2471	0.73	14. Repetitive CNV	1.56
	**CD34+ > AML**							15. Repetitive CNV	2.67
							12. Repressed Chromatin	2.14
							42. HAIBMethylRRBS sites Rep2	1.95
>0.99		4.35%	698394	1278	193	0.87	41. HAIBMethylRRBS sites Rep1	1.94
>0.95		12.20%	393652	3610	961	0.83	35. Histone H3K27me3	1.87
>0.90		17.46%	281713	5264	1921	0.84	47. cancer SNV AML	1.8
							14. Repetitive CNV	1.79
							43. CpG Islands	1.6
							3. Poised Promoter	1.5
**AML > CD34+**								
	< 0.01	3.91%	446827	545	271	1	47. cancer SNV AML	1.92
	< 0.05	11.72%	268043	2030	1354	1.03	44. vistaEnhancers	1.86
	< 0.10	17.52%	200314	3400	2707	1	50. AML chromosome translocations	1.72
	**Granulocyte > AML**								
>0.99		5.77%	931140	1362	193	0.81	15. Repetitive CNV	2.62
>0.95		15.31%	493223	3057	965	0.71	47. cancer SNV AML	1.67
>0.90	21.01%	338224	4133	1931	0.71	14. Repetitive CNV	1.66
**AML > Granulocyte**							12. Repressed Chromatin	2.64
							35. Histone H3K27me3	2.31
	< 0.01	3.84%	484980	746	245	1.23	47. cancer SNV AML	1.78
	< 0.05	11.20%	281623	2953	1232	1.17	37. histone H3K9me1	1.74
	< 0.10	16.81%	211270	4959	2465	1.13	3. Poised Promoter	1.69
							7. WeakEnhancer	1.6
							19. DNase Clustered	1.53

Chromosomal domains with strongest changes in H3K9me2 Sum Log Ratio were determined for the six comparative categories of H3K9me2 dLOCKs: high in CD34+ vs. granulocyte (CD34+ > Granulocyte); high in granulocytes vs. CD34+ (Granulocyte > CD34+); high in CD34+ vs. AML cluster A (CD34+ > AML); high in AML cluster A vs. CD34+ (AML > CD34+); and high in granulocytes vs. AML A (CD34+ > AML); high in AML cluster A vs. granulocytes (AML > Granulocytes). Percent of the human genome, average LOCK size, total number of genes in dLOCKs, total number of dLOCKs, and average genes/dLOCKs are shown for each category with alpha > 0.90, 0.95, 0.99 and alpha < 0.10, 0.05, 0.01. Selected genomic features enriched more than 1.5 fold are shown for each category with alpha > 0.99 and alpha < 0.01. For genomic references (as numbered) and other enrichment data see Materials and Methods and **S6 Table**.

**Fig 5:**
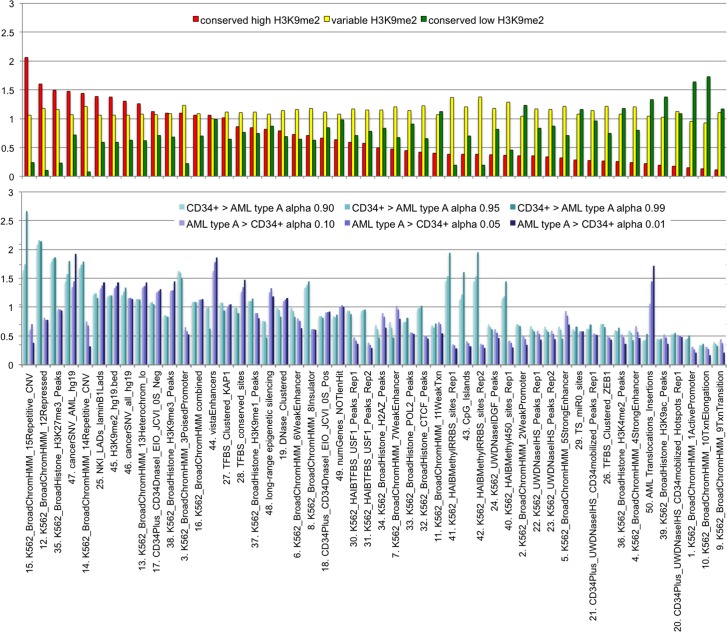
Major genomic features associated with genes located within H3K9me2 domains altered during myeloid differentiation and AML. Associations (fold enrichment) of selected genomic features within differential H3K9me2 domains altered in myeloid cells. The top panel shows: domains of constant high (red) variable (yellow) and constant low (green) H3K9me2 in all myeloid cells; The bottom panel shows: H3K9me2 dLOCKs maximal and minimal in CD34+ vs. AML cluster A. Data in the top panel are based on dLOCKs at alpha>0.90, 0.95, 0.99 and dLOCKs at alpha<0.10, 0.05, 0.01). Data in the bottom panel are based on dLOCKs at alpha >0.95 and <0.05. For references (as numbered) see Materials and Methods and **S6 Table**).

Our analysis revealed extended chromosomal domains with average size range between approximately 200 and 900 Kb. Despite the overall genome-wide correlation of H3K9me2 domains with low gene density (**Fig 2**), the dLOCKs did not show a substantial depletion in the gene density (**Table 1** and **Fig 5**). Analysis of dLOCK gene intersections between the normal and AML cells helped us to answer the question whether the genome-wide changes in H3K9me2 merely reflect an interrupted differentiation process in AML or they include some genes showing gains or loss of H3K9me2 specific for the disease state. We found that, contrary to the former hypothesis that would predict complete intersection of the dLOCKs changed in the disease with those changed during myeloid differentiation, just ~38% of genes from the dLOCK^CD34+>AMLA^ did intersect with dLOCK^CD34+>granulocyte^ while ~51% of genes from dLOCK^CD34+>AMLA^ intersected with dLOCK^granulocyte>AML^. Reciprocally, only ~32% of genes from dLOCK^AMLA>CD34+^ intersected with dLOCK^granulocyte>CD34+^ while ~40% of genes from dLOCK^AMLA>CD34+^ intersected with dLOCK^AML>granulocyte^ (**Fig H in S1 File**). We thus conclude that AML cells, exhibit large-scale alterations (both gains and losses) in the levels of H3K9me2 that are either specific to the disease state or are transiently forming during some early pre-leukemia differentiation stages and preserved in AML.

### Large blocks of H3K9me2 show AML-specific variations in genomic features related to gene regulation and genomic stability

The observation of the conserved and variable blocks of H3K9me2 (**Fig 2**) prompted us to analyze their selective enrichment with specific genomic features. First, we designated the conserved high (“Red”), transient (“Yellow”), and conserved low (“Green”) H3K9me2 zones by calculating the standard deviation of the 10 kb window scores of H3K9me2 in the all myeloid samples combined (see Materials and Methods). The Red, Yellow, and Green zones occupy 12.2%, 66.4% and 21.4% of the human genome respectively. For these genomic zones as well as for each of the six categories of dLOCKs, we calculated the enrichments of the genomic features from UCSC/ENCODE databases (**Fig 5 and S6 Table**).

Consistent with their repressed state, the Red H3K9me2 domains are depleted in all “active chromatin” traits and are notably enriched (≥1.5 fold) in just a few genomic features: repetitive copy number variations (CNV), repressed chromatin and histone H3K27me3 (**Fig 5**). Among the dLOCKs, the strongest difference between the CD34+ progenitors and AML is found for the “repressed” chromatin and H3K27me3 (**Fig 5**), in agreement with the genome-wide correlations (**Fig 3**). These epigenetic marks are strongly (more than 2-fold) enriched in dLOCK^CD34+>AML A^ and depleted in dLOCK^AML A>CD34+^ (**Table 1 and Fig 5**). We also observed a notable enrichment of dLOCK^CD34+>AMLA^ for repetitive copy number variations (CNVs). Since the last result could potentially reflect changes in DNA copy numbers rather than ChIP, we additionally analyzed the DNA annotated as repetitive CNV among our samples and observed no significant variation among all primary cells with exception of increased copy numbers in K562 (**Fig I in S1 File).**

The prominent specific features of the H3K9me2 blocks enriched in AML, as compared to those enriched in CD34+, are the depletion of DNA methylation sites and density of CpG islands as well as the enrichment with vista enhancers (**Fig 5**). These findings suggest that the loss of histone H3K9me2 dLOCK^CD34+>AMLA^ could cause de-repression of genes controlled by DNA methylation in AML. In contrast gain of H3K9me2 at vista enhancers within dLOCK^AMLA>CD34+^ would cause repression of corresponding genes in AML.

The most universally conserved genomic features enriched in all six types of the H3K9me2 dLOCKs are the AML SNV densities confirming the association of H3K9me2 with regions of increased mutagenesis, apparently due to inhibited DNA repair in heterochromatin [62]. In contrast, the enrichment of genes involved in chromosomal translocations known to cause AML (S3 **Table**) was prominent only in dLOCK^AMLA>CD34+^ (**Table 1 and Fig 5**) suggesting that, in addition to transcriptional regulation, the rearrangement of large blocks of H3K9me2 in acute myeloid leukemia is linked to chromosomal translocations and genomic instability.

### AML-specific blocks of H3K9me2 contain repeated and coordinately regulated sets of genes sharing common upstream transcriptional regulators

Gene ontology analysis using Ingenuity® pathway search showed that the differentiation-related and the AML-related dLOCKs were moderately (1.7–2.7 fold) but very significantly (p<10^−6^ –p<10^−15^) enriched with genes listed under disease or function annotation “acute myeloid leukemia” (**Fig H in S1 File**). The most prominent among the H3K9me2 blocks depleted in AML A (dLOCK^CD34+>AMLA^) are gene-rich clusters containing multiple repeats of *ZNF* genes (KRAB-ZFP, zinc finger proteins containing a Kruppel-associated or KRAB domain [63]) genes on chromosome 19 and elsewhere in the genome **(Fig 4** and the top lanes in **Tables C** and **E in S5 Table).** Other examples of large repeated gene families within the dLOCKs depleted with H3K9me2 in AML include repeats of small nucleolar RNA SNORD116 and SNORD115 on chromosome 15 and clusters of protocadherin genes on chromosome 5 (lanes 12 and 32 in **supp. Table E in S5 Table)**.

In contrast to the AML-depleted dLOCKs, the AML—gained dLOCKs, (dLOCK^AMLA>CD34+^ and dLOCK^AMLA>Gran^) are not enriched with repeated genes and have a smaller magnitude of H3K9me2 variations **(Tables D and F in S5 Table**)**.** Remarkably, they contain unique genes associated with hematological development and AML such as *RUNX1*, *ETV6*, *ERG*, *MECOM* and *ETS2* among other genes (**Table B in S7 Table**).

**Fig 6:**
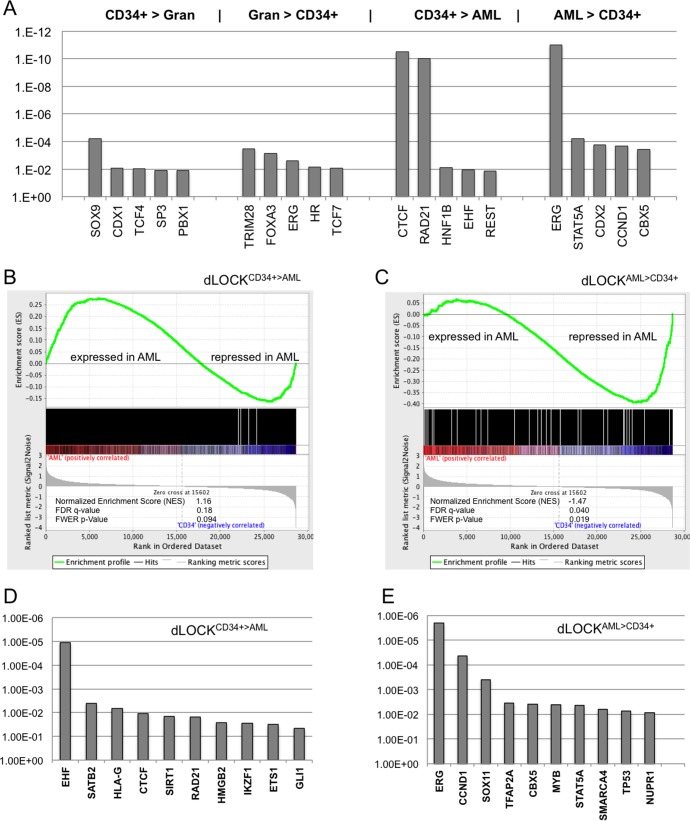
Genes within the transient H3K9me2 blocks are controlled by specific upstream regulators and undergo massive transcriptional changes in AML. A: Top upstream transcriptional regulators associated with total genes within the four types of dLOCKs (a>0.95; a<0.05) shown at the top. B, C: Gene set enrichment analysis [64] with AML and CD34+ control cell populations reveals contrasting association of the H3K9me2 dLOCK^CD34+>AML^ (B) and AML-repressed genes within dLOCK^AML>CD34+^ (C) with genes up- and down-regulated in AML. D, E: Top upstream regulators associated with the AML-expressed genes within dLOCK^CD34+>AML^ (D) and AML-repressed genes within dLOCK^AML>CD34+^ (E).

We have used Ingenuity® pathway analysis to identify upstream transcription regulatory molecules associated with coordinated changes in the dLOCKs (alpha < 0.05 and alpha >0.95). We found the regulatory molecule ERG standing at the top within the H3K9me2 blocks enriched in AML (dLOCK^AMLA>CD34+^, *p*<10^−11^). In contrast, in the H3K9me2 blocks depleted in AML the most significant upstream regulators (*p*<10^−10^) were CTCF and RAD21 (cohesin), the transcriptional factors that also regulate chromosome domain boundaries. TRIM28 (KAP-1)**,** a common co-repressor of *KRAB-ZFP* (*ZNF*) genes was prominent in the H3K9me2 blocks enriched in granulocytes (**Fig 6A**). Thus, our data show that analysis of genes within differential H3K9me2 domains reveals specific regulatory pathways affected by large-scale epigenetic changes in AML.

### AML-enriched blocks of H3K9me2 are associated with massive gene silencing

To examine whether the genome-wide changes in histone H3K9me2 domains correspond to altered expression in the underlying genes, we conducted gene expression array experiments in AML cluster A cells and normal CD34+ bone marrow stem cells (chosen as the normal cells most similar to AML cluster A in their genome-wide H3K9me2 distribution (**Fig 3B**)). mRNA expression was assayed using Illumina HumanHT-12 array containing 33,732 hybridization probes with distinct expressed sequence tags. Of these, 7346 transcription units showed significant changes (p<0.05) between AML and control (CD34+) samples (**Table C in S7 Table**).

We used Broad Institute gene set enrichment analysis, GSEA, [64] to determine associations between the genes from the CD34+/AML gene expression arrays and genes within the transient H3K9me2 blocks in AML and CD34+ cells. GSEA revealed a dramatic difference between the enrichment of AML-activated genes in dLOCK^CD34+>AML^ and enrichment of AML-repressed genes in dLOCK^AML>CD34+^ (**Fig 6B and 6C and Table D in S7 Table**), suggesting that transcriptional repression of multiple genes in AML is associated with the AML-enriched H3K9me2 blocks. For the top-scoring genes associated with dLOCK^CD34+>AML^ and upregulation in AML, Ingenuity® pathway analysis of upstream transcriptional activators revealed several transcriptional regulators including CTCF and RAF21 (**Fig 6D**) but not TRIM28 apparently because, although its target *KRAB-ZFP* genes are enriched in H3K9me2, they are known to resist silencing by G9a/GLP [17].

Remarkably, among the top-scoring transcriptional regulators associated with dLOCK^AMLA>CD34+^ and downregulation in AML (**Fig 6E**) we found ERG as the top transcriptional regulator (P<10^−5^) just as it was prominent in the total dLOCK^AMLA>CD34+^ genes (**Fig 6A**). ERG expression and ERG-regulated pathways are associated with stem cell characteristics and poor prognosis of AML [65, 66]. We thus concluded that the ERG-regulated genes associated with dLOCKs and silenced in AML are the most likely to be regulated by H3K9me2 and to fulfill functions contributing to AML progression.

### Reversing of the H3K9me2 blocks and associated gene silencing by G9a inhibition

**Fig 7:**
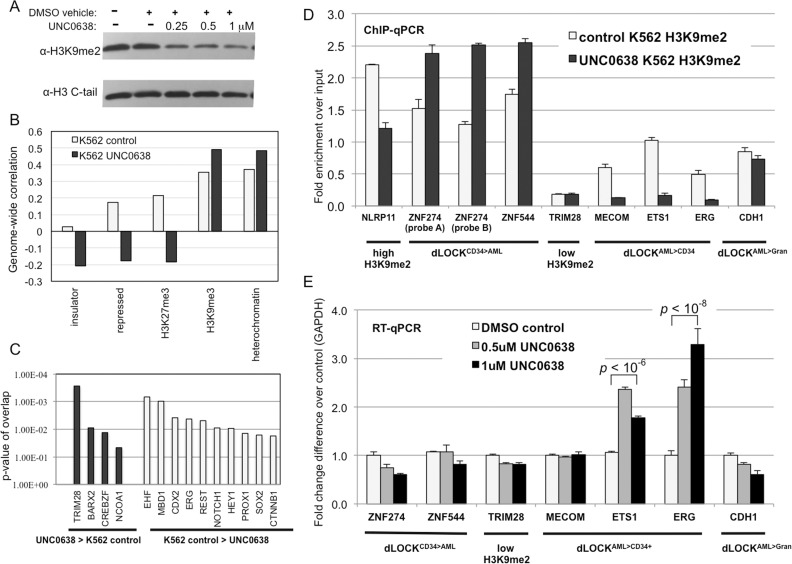
Inhibition of G9a/GLP in K562 decreases H3K9me2 levels and activates genes within AML-enriched transient H3K9me2 blocks. A: Western blotting probed with antibodies against H3K9me2 and unmodified histone H3 show decreasing levels of H3K9me2 in K562 cells treated with G9a/GLP inhibitor UNC0638. B: Graphs showing correlations between H3K9me2 domains in control and 1 μM UNC0638-treated K562 cells vs. selected biodata with top discriminatory power (insulator, repressed chromatin, H3K27me3, H3K9me3, and heterochromatin). C: Ingenuity pathway analysis showing upstream regulators associated with the dLOCK^UNC0638>K562control^ and dLOCK^K562control>UNC0638^. D: H3K9me2 levels determined by ChIP-qPCR in control and UNC0638-treated K562 cells for selected gene probes representing dLOCK^CD34+>AML^ (*ZNF274*, *ZNF544*), constitutively high H3K9me2 (*NLRP11*), constitutively low H3K9me2 (*TRIM28*), dLOCK^AML>CD34+^ (*MECOM*, *ETS1*, *ERG*) and dLOCK^AML>Gran^ (*CDH1*). E: RT-PCR analysis of gene expression level in control and UNC0638-treated K562 cells for selected gene probes representing dLOCK^CD34+>AML^ (*ZNF274*, *ZNF544*), constitutively low H3K9me2 (*TRIM28*), dLOCK^AML>CD34+^ (*MECOM*, *ETS1*, *ERG*) and dLOCK^AML>Gran^ (*CDH1*). *p–*values represent Student’s t-test for 2 tailed, unpaired equal variance.

Since G9a/GLP, the histone methyltransferase specific for H3K9me2, is involved in repression of several genes associated with AML [24, 25] and contributes to AML progression [26], we asked if we could reverse formation of AML-specific H3K9me2 blocks in human myeloid cells using a pharmacological G9a inhibitor UNC0638 [67]. First, we treated K562 cells with increasing concentrations of UNC0638 and observed a substantial (>2-fold) decrease in total H3K9me2 level by western blot analysis (**Fig 7A**). We then conducted H3K9me2 ChIP-seq analysis of UNC0638-treated and control K562 cells as we did for the other myeloid cells. Remarkably, analysis of genome-wide correlations showed a dramatic decrease in correlation with insulators, repressed chromatin, and H3K27me3 induced by UNC0638 (**Fig 7B**) and recapitulating the trend observed during terminal myeloid differentiation (**Fig 3A**). Accordingly, analysis of upstream transcriptional regulators revealed TRIM28 and ERG among the top transcriptional regulators in dLOCK^UNC0638>K562^ and dLOCK^K562>UNC0638^ respectively (**Fig 7C**).

Next, we tested expression of selected genes residing within the transient H3K9me2 blocks by RT-qPCR in UNC0638-treated and control K562 cells. We chose genes belonging to the two regulatory pathways: TRIM28-regulated *KRAB-ZFP* (*ZNF*) genes most represented in dLOCKs depleted in AML but not reduced by G9a inhibition and ERG-regulated genes (*ERG*, *ETS1*, *MECOM*) residing in the dLOCKs gained in AML and strongly reduced by G9a inhibition in K562 cells (**Fig 7D**). We found that transcription of *KRAB-ZFP* genes was not affected by G9a inhibition consistent with previous findings [17]. Furthermore, *MECOM* gene was also not affected despite a significant loss in H3K9me2 thus showing that reduction of H3K9me2 *per se* is not sufficient to reverse repression of all silent genes in dLOCKs. Probably, this is due to some additional levels of epigenetic regulation, such as DNA methylation, could be established upon histone methylation [68] to prevent gene reactivation. In contrast, ERG-regulation pathway genes, *ERG* and *ETS1*, were significantly (*p* < 10^−8^ and <10^−6^ respectively) activated by UNC0638 treatment (**Fig 7E**). Since both of these genes are involved in AML leukemogenesis [69–71], we concluded that G9a/GLP histone methyltransferases could have an important role in restricting expression of these oncogenes by forming the AML-specific H3K9me2 blocks. Furthermore, since ERG is the top upstream regulator of the genes within the AML-enriched blocks (**Fig 6A**) and is itself downregulated by G9a (**Fig 7E**), our finding indicates that ERG may act as a part of a regulatory circuit in which its repression by G9a/GLP may lead to a decreased expression of downstream genes positively regulated by ERG and negatively by G9a/GLP-mediated H3K9me2.

## Discussion

We have mapped extensive epigenomic domains of histone H3K9 dimethylation in myeloid cells. As expected, the overall genome-wide distribution of H3K9me2 was consistent with the large genomic blocks (LOCKs) previously described in non-myeloid cells [5, 18] and showed a strong positive correlation with H3K9me2 [52] and LADs [46, 60] in non-myeloid human cells and a negative correlation with gene density (**Fig 2**). Also as expected was the correlation of the myeloid H3K9me2 domains with somatic cancer mutations [53] and AML-specific SNV [11]. Remarkably, H3K9me2 showed significantly stronger correlation with LADs than the two other repressive heterochromatin marks, H3K9me3 and H3K27me3 in the human genome database (**Fig 2**). Correlation of H3K9me2 within different myeloid and non-myeloid samples was dramatically stronger than that between H3K9me2 vs. H3K9me3 and H3K27me2. The H3K9me2 domains in myeloid cells are thus distinct from other repressive heterochromatin marks and may play a unique role in spatial organization of the condensed facultative heterochromatin during terminal granulocytic differentiation, and inhibit chromatin access to the DNA repair processes in proliferating cells.

**Fig 8:**
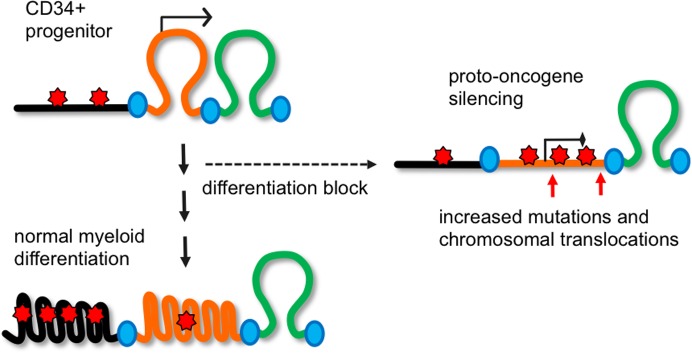
A proposed role of the transient blocks of H3K9me2 in transcriptional repression and chromosomal instability in myeloid leukemia. During normal myeloid differentiation, H3K9me2 marks (red stars) accumulate on heterochromatic chromosomal domains (black) that, together with developmentally- repressed facultative heterochromatin (orange) form condensed heterochromatin blocks (curved lines). At the onset of myeloid leukemia, the transitional blocks of H3K9me2 containing proto-oncogenes involved in stem cell maintenance in CD34+ progenitors (orange) accumulate H3K9me2 and become transcriptionally silenced. These transitional blocks are prone to increased mutagenesis and chromosomal translocations. The proto-oncogenes within the H3K9me2 blocks are likely to become reactivated as a result of the boundary site mutations or chromosomal translocations disrupting the chromatin boundaries (blue ovals) between the inactive and constitutively active (green) domains and leading to more aggressive forms of acute myeloid leukemia.

In our previous works, we observed that H3K9me2 modification marks the relaxed chromatin in the nuclei of proliferating cells and the condensed facultative heterochromatin in the nuclei of terminally differentiated cells [19–21, 32]. Here we show that H3K9me2 was alternatively associated with two distinct chromatin states—the repressed euchromatin prevailing in CD34+ progenitors and heterochromatin in terminally differentiated granulocytes. This transition is consistent with facultative heterochromatin marked by H3K9me2 merging with constitutive heterochromatin marked by H3K9me3 and undergoing mutual condensation during terminal myeloid differentiation in granulocytes (**Fig 8**). Surprisingly, in AML cluster B as well as in leukemic K562 cells, there are comparably high levels of H3K9me2 mark at heterochromatin which, however, does not lead to an apparent chromatin condensation. This finding suggest that some developmentally-regulated factors, absent in the nuclei of AML and K562 cells, are needed to condense the facultative heterochromatin during terminal myeloid differentiation. For example, the nuclear serpins MENT (serpin B10) and MNEI (serpinB1) accumulate in the nuclei of differentiated chicken [72] and human [19, 32] granulocytes in association with condensed heterochromatin though the mechanism of their interaction with H3K9me2 remains to be understood.

During normal cell differentiation, H3K9me2 is significantly increased and spreads over repressed chromosomal domains as shown for differentiating mouse lymphocytes [73] and stem cells [18] including hematopoietic progenitors [17]. Furthermore H3K9methylation (both di- and tri-methylation) and HMTases G9a/GLP and SetDB1 are known to inhibit pluripotent stem cell features [4, 74] suggesting that spreading of H3K9me2 would promote myeloid differentiation and restrict AML progression. More recently, however, genetic inactivation of G9a/GLP in a mouse model as well as pharmacological inhibition in human cells was shown to slow down AML proliferation [26] suggesting that in leukemic stem cells G9a/GLP and H3K9me2 may promote self-renewal and proliferation and that the cellular phenotypes determined by G9A/GLP and H3K9me2 are highly context dependent.

One of the prominent differences between the H3K9me2 blocks enriched in AML as compared to those enriched in CD34+, are the depletion of DNA methylation sites and CpG islands (**Fig 5**). CpG islands were reported as the sites most strongly overlapping with H3K9me2 peaks in CD34+ hematopoietic progenitors [17] while many leukemia-deregulated genes are associated with the areas of normally low DNA methylation (“canyons”) [75]. Our finding thus suggest that the massive rearrangement of histone H3K9me2 in AML could cause de-repression of genes controlled by CpG methylation in normal progenitors and/or repression at the aberrant DNA methylation loci normally residing in the DNA methylation canyons.

Our identification of the AML types with striking differences in H3K9me2 levels at dLOCKs makes it necessary to understand for which particular AML cases a pharmacological reversal of H3K9me2 domains and associated transcriptional changes could benefit or interfere with AML therapy? Here we observed that inhibition of G9a/GLP suppresses AML-specific blocks of H3K9me2 and leads to a moderate activation of the embedded proto-oncogenes *ERG* and *ETS1* (**Fig 7**). The increased expression of ERG promotes hematopoietic stem cell maintenance [30], is linked to a poor outcome of AML [65, 66], is associated with chemoresistance in leukemia [70], and thus is likely to have an adverse effect on AML therapy. Interestingly, a recent work has reported a notable decrease of chromatin accessibility at a number of key regulatory genes in AML pre-leukemia stem cells [76].

At present, our study is limited by using one myeloblastic cell line, K562, derived from chronic myeloid leukemia, and total samples of CD34+ stem cells and progenitors isolated from bone marrow. While our leukemic samples were derived from peripheral blood, an alternative sources of control blood progenitors such as mobilized blood CD34+ cells or cord blood-derived CD34+ cells are also not ideal because their collection requires treatment with G-CSF [34], a cytokine which has significant cellular stimulatory effects or represents fetal tissues which developed only prenatally. The leukemia cells under study all originate from adult bone marrow stem or progenitor cells, thus we consider the marrow CD34+ cells to be biologically closest to the studied leukemias and thus optimal control cells. Future experiments on H3K9me2 mapping and G9a/GLP inhibition including functional tests for stem cell characteristics in primary and cultured AML-derived cells (such as KG-1 or HL-60) as well as analysis of homogeneous populations of pre-leukemic stem cells are needed to reveal the role of heterochromatin in repressing the proto-oncogenes and associated stem cell features in AML leukemia and pre-leukemia states.

We also noted that among the LOCKs specific for AML, there is a notable enrichment for sites that undergo chromosomal translocations in AML as well as for AML SNVs (**Table 1).** This could result from the developmentally-regulated spreading of heterochromatin that may promote subsequent mutagenesis by inhibiting DNA damage repair [62, 77]. Recently, it had been shown that many proto-oncogenes reside in insulated chromosomal neighborhoods and often become activated by chromosomal translocation or boundary site mutations [78]. Therefore, spreading of H3K9me2 blocks onto a number of proto-oncogenes (*RUNX1*, *RUNX1T1*, *MECOM*, *ERG*, *ETV6*, and others) at the onset of leukemogenesis may create favorable conditions for their abrupt and highly oncogenic activation by chromosomal translocations (**Fig 8**). Accumulation of large number of myeloblasts with high level of H3K9me2 at the proto-oncogenes in AML is likely to sharply increase the overall rate of mutagenesis and translocations and, therefore, the chance of AML acceleration and/or relapse. Which primary events (mutations and/or epigenetic changes) lead to the spreading of H3K9me2 blocks remains to be determined. However, our observation that the H3K9me2 blocks can be suppressed by pharmacological inhibition of the histone methyltransferase G9a/GLP (**Fig 7**) suggests that limiting H3K9me2 spreading in AML may prevent the reactivation of oncogenes resulting from the secondary AML-specific gene mutations and chromosomal translocations. Therefore, our approach to monitoring H3K9me2 levels at proto-oncogenes and regions of genomic instability may become instrumental in testing new G9a inhibitors more efficient in slowing down AML cells growth and at the same time reducing mutagenesis and preventing secondary mutations in AML. To make “epigenetic therapy” of AML and other myeloid disorders most efficient, we need to understand the fine balance within the heterochromatin-mediated mechanisms that simultaneously affect both gene expression and chromosomal stability at specific chromosomal locations during myeloid differentiation.

## Supporting information

S1 File Combined supporting figures. This file contains nine figures labeled A to I.
(PDF)

S1 Table List of AML samples. (XLSX)

S2 Table List of sequence data sets and GEO submission entries. (XLSX)

S3 Table List of genes undergoing chromosome translocations in AML. (XLSX)

S4 Table Genome-wide correlations. (XLSX)

S5 Table dLOCKs. This file contains ten tables labeled A–J.
(XLSX)

S6 Table dLOCK enrichments versus genome averages. (XLSX)

S7 Table Gene sets. This file contains ten tables labeled A–D.
(XLSX)

S8 Table PCR primers. (DOCX)
