# Supplementary material for: Genome-wide mapping of histone H3K9me2 in acute myeloid leukemia reveals large chromosomal domains associated with massive gene silencing and sites of genome instability
Source: PLoS One. 2017 Mar 16;12(3):e0173723. doi: 10.1371/journal.pone.0173723 (PMC5354391; doi:10.1371/journal.pone.0173723)
Supplement: S1 File — This file contains nine figures labeled A to I. (PDF) [file pone.0173723.s001.pdf]

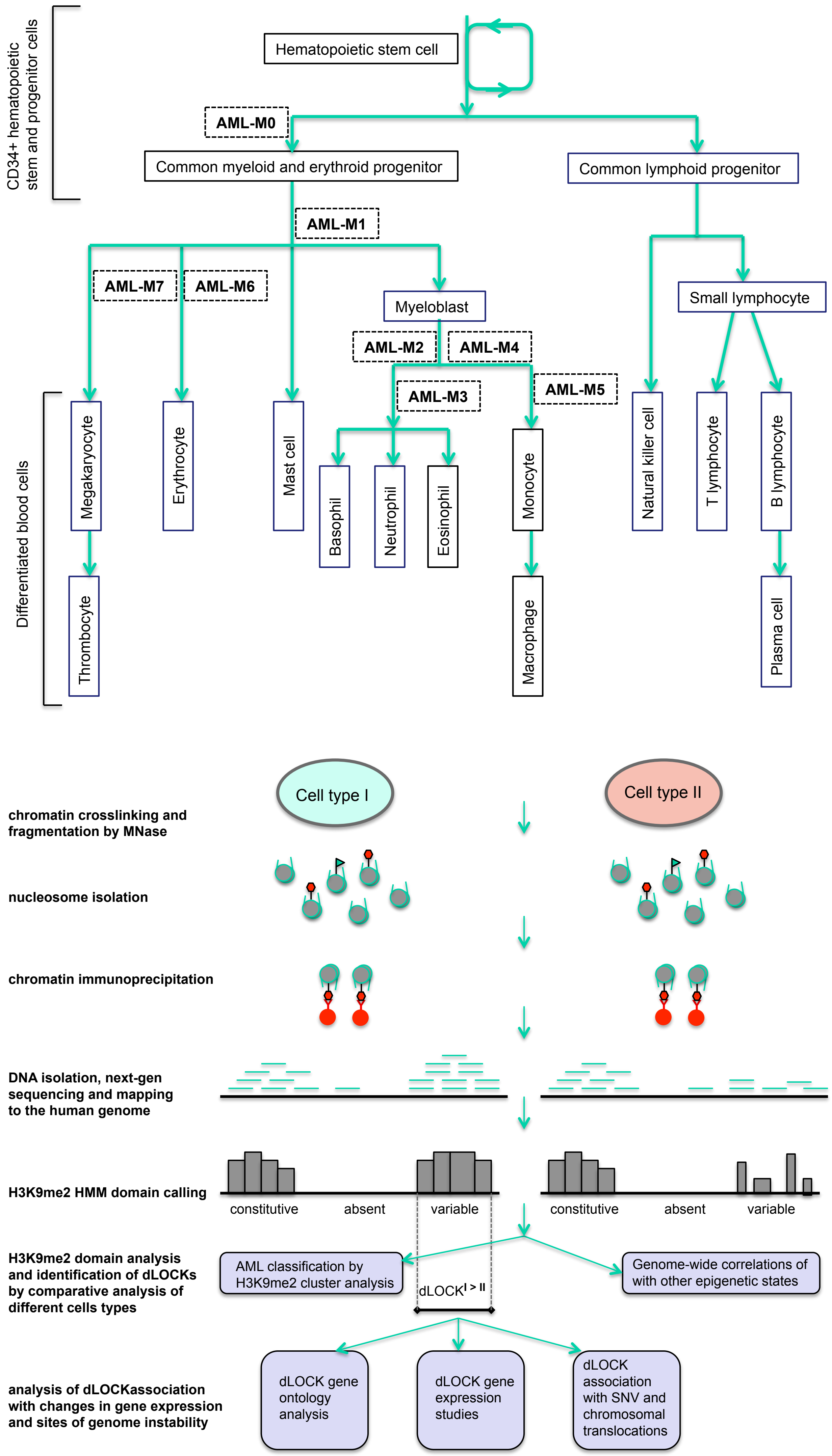

Fig A. Flowchart for genome-wide analysis of H3K9me2 domains in myeloid and AML cells.
Top: schematic of human hematopoietic differentiation showing main types of undifferentiated hematopoietic progenitor and stem cells and differentiated blood cells (solid boxes). Different type of AML (dashed boxes, FAB classification) emerge at different stages of myeloid differentiation. **Bottom:** diagram of analyses performed for identifying dLOCKs different between any two myeloid cell types (designated here as type I and type II).

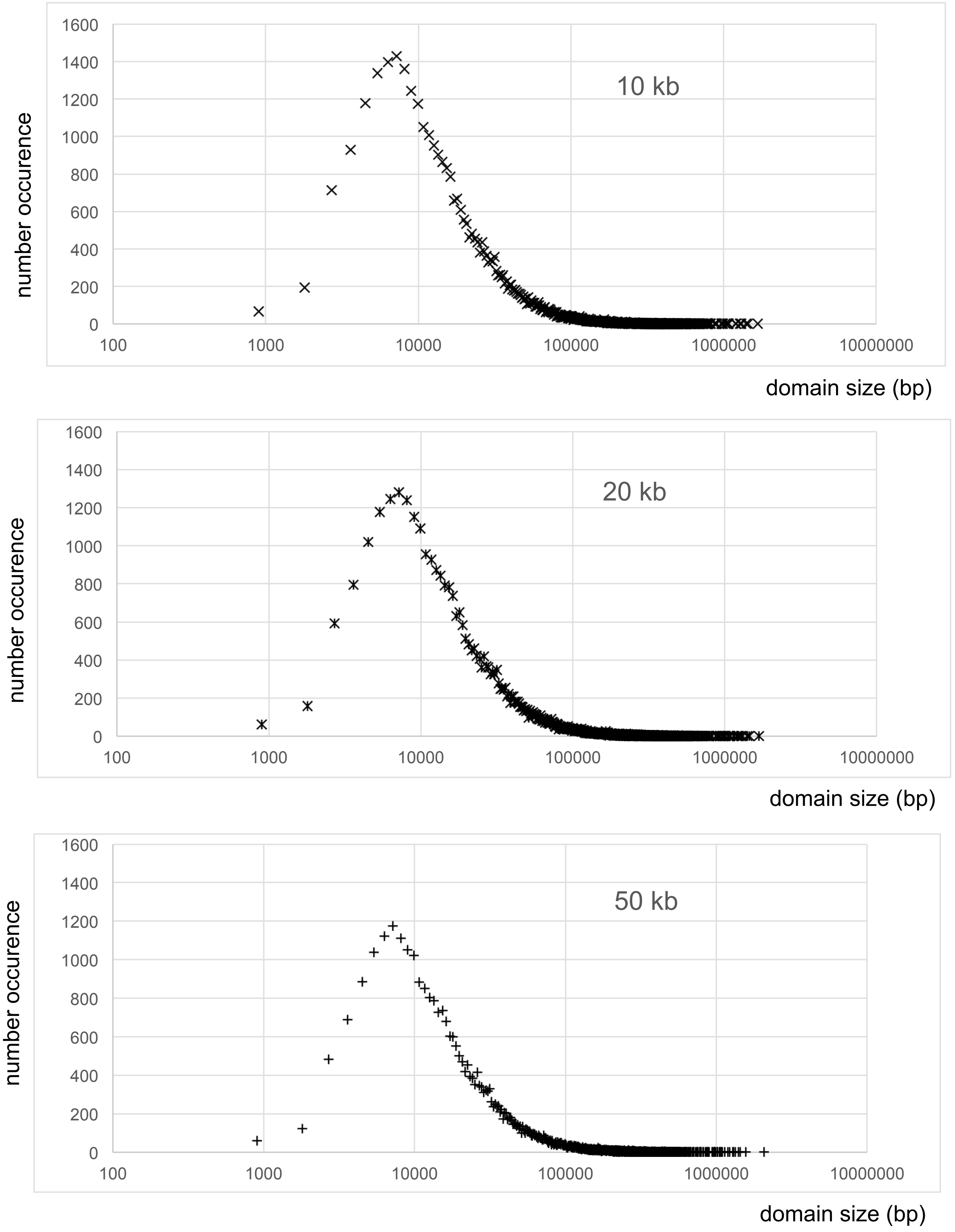

Fig B. RSEG domain size distributions.

Histograms showing size distributions of RSEG domains (H3K9me2 in K562 cells) calculated for predicted value of 10 kb (top), 20 kb (middle), and 50 kb (bottom).

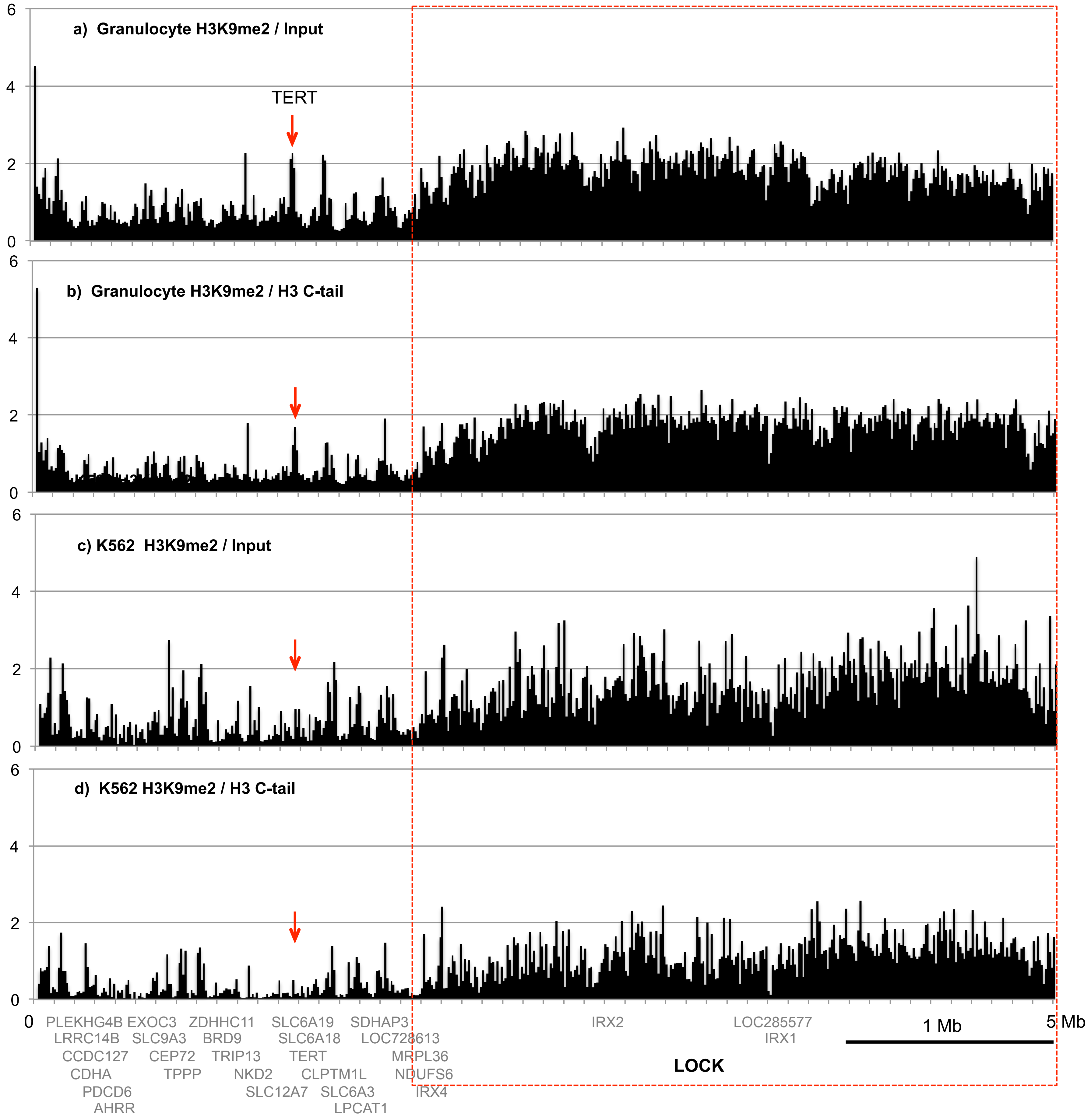

Fig C. Mapping of H3K9me2 normalized to DNA input and control ChIP with unmodified histone H3 C-tail antibody. (a-d): distribution of DNA sequence reads mapped to the human genome at 10 kb resolution (showing a region of chromosome 5 between 0 and 5 Mb). DNA sequence reads were derived from H3K9me2 ChIP-seq of granulocytes (a, b) and K562 cells (c, d). The reads were grouped by 10 kb windows (see Suppl. Table S3) and normalized to granulocyte input DNA (a), granulocyte ChIP-seq of H3 C-tail (b), K562 input DNA (c), and K562 ChIP-seq of H3 C-tail (d). The H3K9me2 LOCK (red dashed box) and the human TERT gene (vertical red arrows) are indicated as in Fig. 1. Y-axes represent fold enrichment of H3K9me2 ChIP-seq sequence reads vs. input DNA (a, c) and H3 C-tail ChIP-seq (b, d).

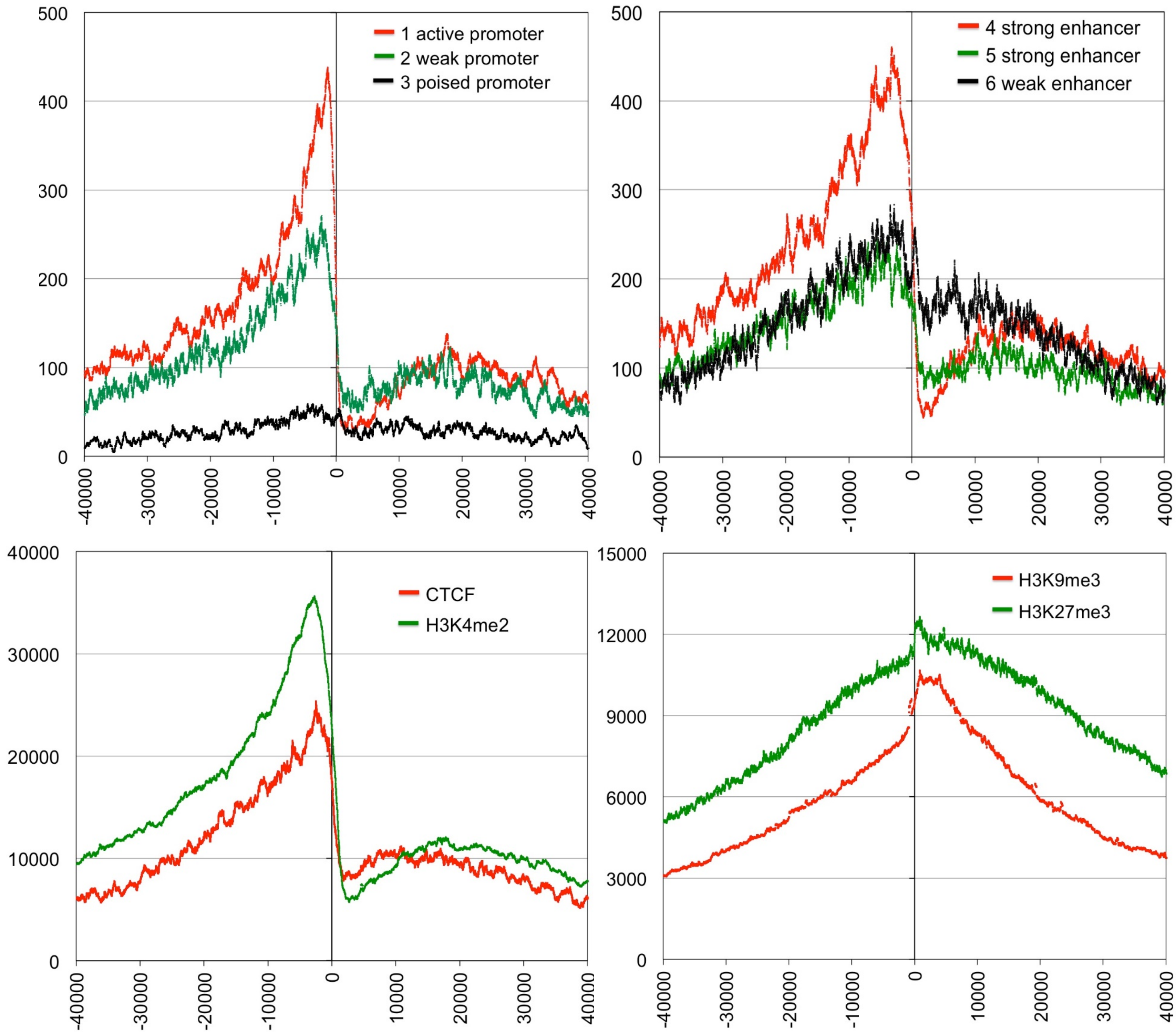

Fig D. Profiles of genomic and chromatin features around H3K9me2 boundaries in K562 cells. Profiles of aligned H3K9me2 HMM derived from K562 H3K9me2 ChIP-seq showing positional correspondence between their boundaries and boundaries of the indicated variables from ENCODE/Broad Institute K562 databases [43]. Genome-wide positions of the analyzed genomic features were converted to coordinates related to the nearest H3K9me2 HMM domain border (positive: outside domain, negative: inside domain). Vertical lines mark H3K9me2 domain boundary positions. Y-axis value is the sum of either a count of 1 per feature for the corresponding x location (i.e. a histogram) for the K562 BroadChromHMM datasets (top two graphs) or it is the sum of the peak enrichment value of each feature in the corresponding x location for the K562 CTCF and Histone datasets (bottom two graphs).

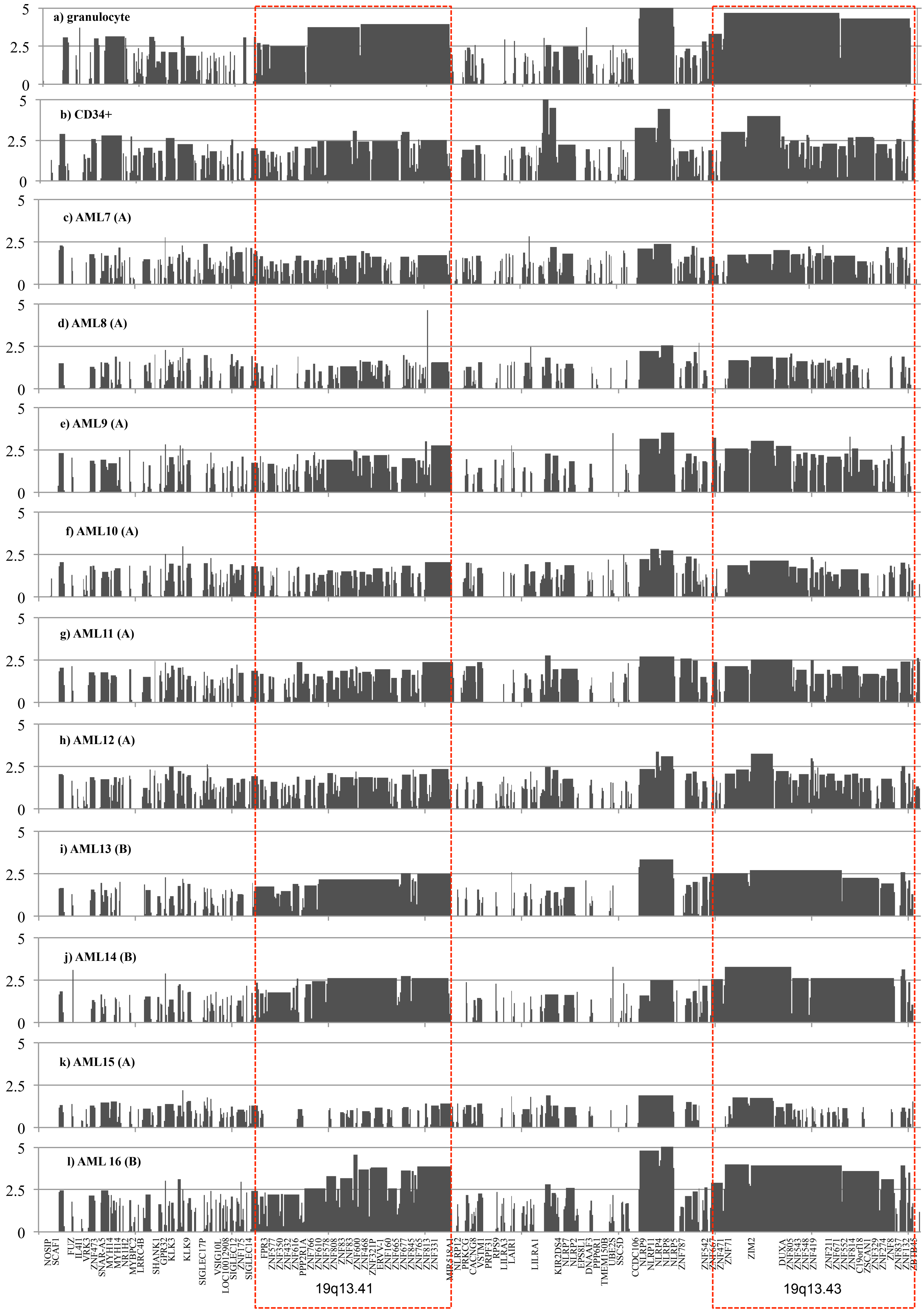

Fig E. Large blocks of H3K9me2 show cell-specific alterations detected by H3K9me2 ChIP and HMM domain mapping.

High resolution maps (10 K windows) of normalized HMM domains on human chromosome 19 locus between 50 and 59.12 Mb showing dLOCKat 19q13.43 (56,940-59,050 kb) dLOCK at 19q.13.41 (52,290-54,280 kb), red dashed boxes. HMM domains were derived from H3K9me2 ChIP-seq of normal granulocyte (a), CD34+ cells (b), and AML7 - AML16 (panels c - l).

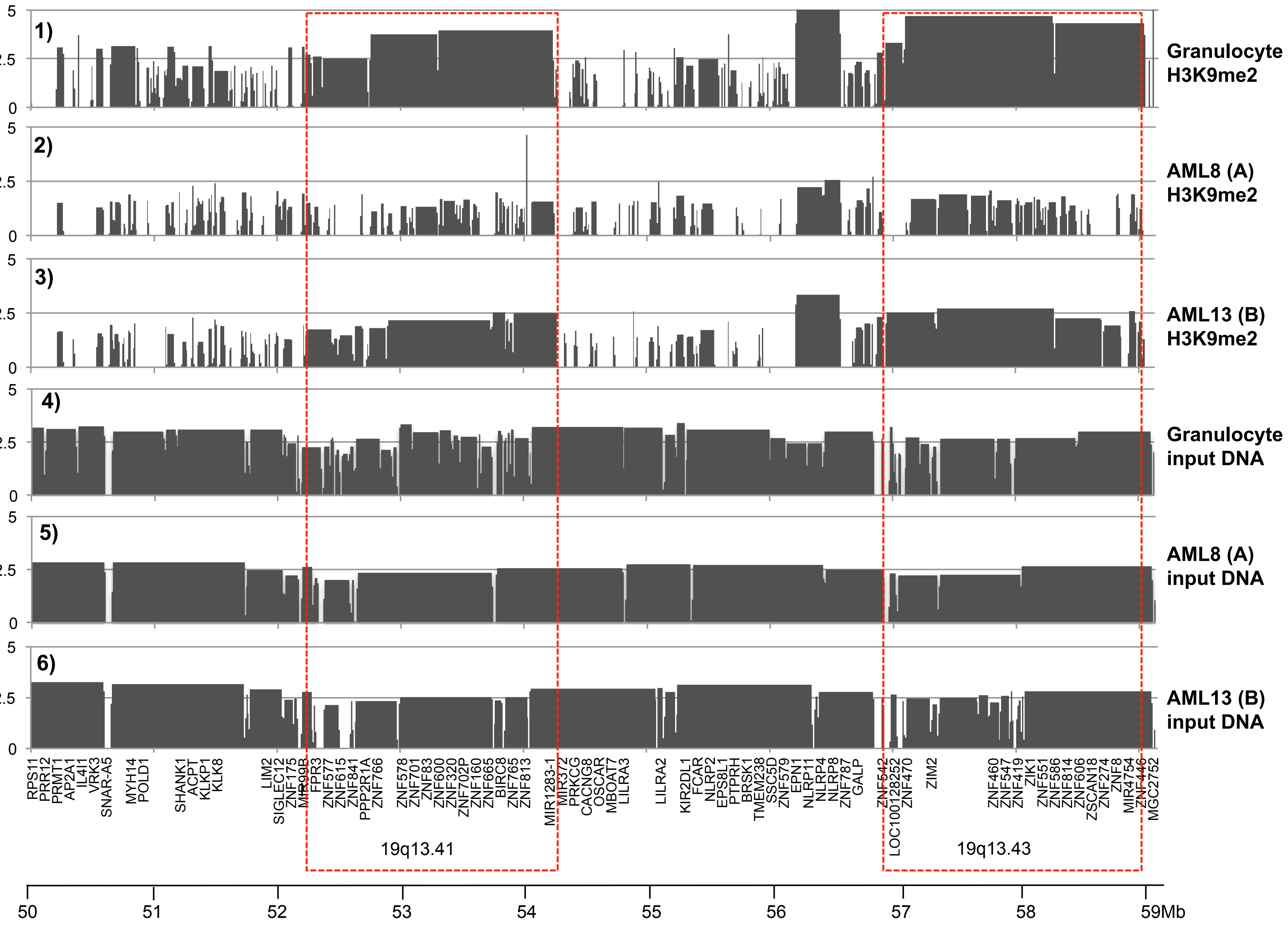

Fig F. Cell-specific alterations of H3K9me2 blocks are detected by ChIP but not by input DNA mapping.

High resolution maps (10 K windows) of normalized HMM domains on human chromosome 19 locus between 50 and 59.12 Mb showing large LOCKs with H3K9me2 low in AML A but not in AML B (19q13.43 and 19q.13.41) marked by dashed boxes. HMM domains were derived from H3K9me2 ChIP-seq of normal granulocyte (1), AML8 (cluster A) (2), AML13 (cluster B) (2), and input DNA of granulocyte (4), AML 8 (5) and AML 13 (6).

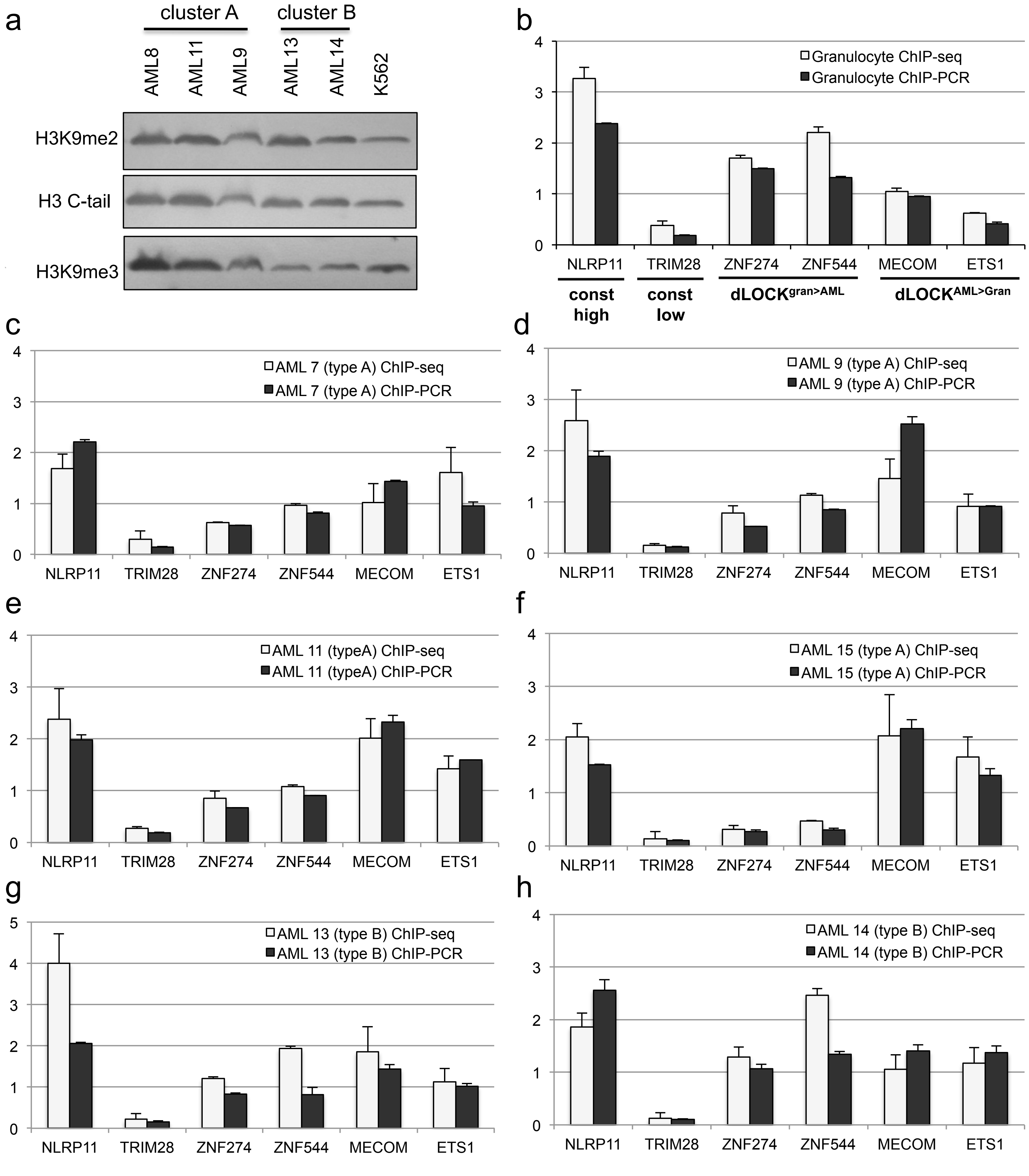

Fig G. Validation of ChIP analysis by genomic qPCR.

a: Western blotting analysis of histone H3K9me2 in samples of AML cluster A and cluster B. **b-h**: comparison of gene-average H3K9me2 levels derived from ChIP-seq with H3K9me2 levels determined by ChIP-qPCR for selected gene probes representing dLOCK$^{gran>AML}$ (ZNF274, ZNF544), constitutively high (red) H3K9me2 domains (NLRP11), constitutively low H3K9me2 domains (TRIM28), and dLOCK$^{AML>gran}$ (MECOM, ETS1) for granulocytes (b) AML cluster (c-f) and AML cluster B (g, h).

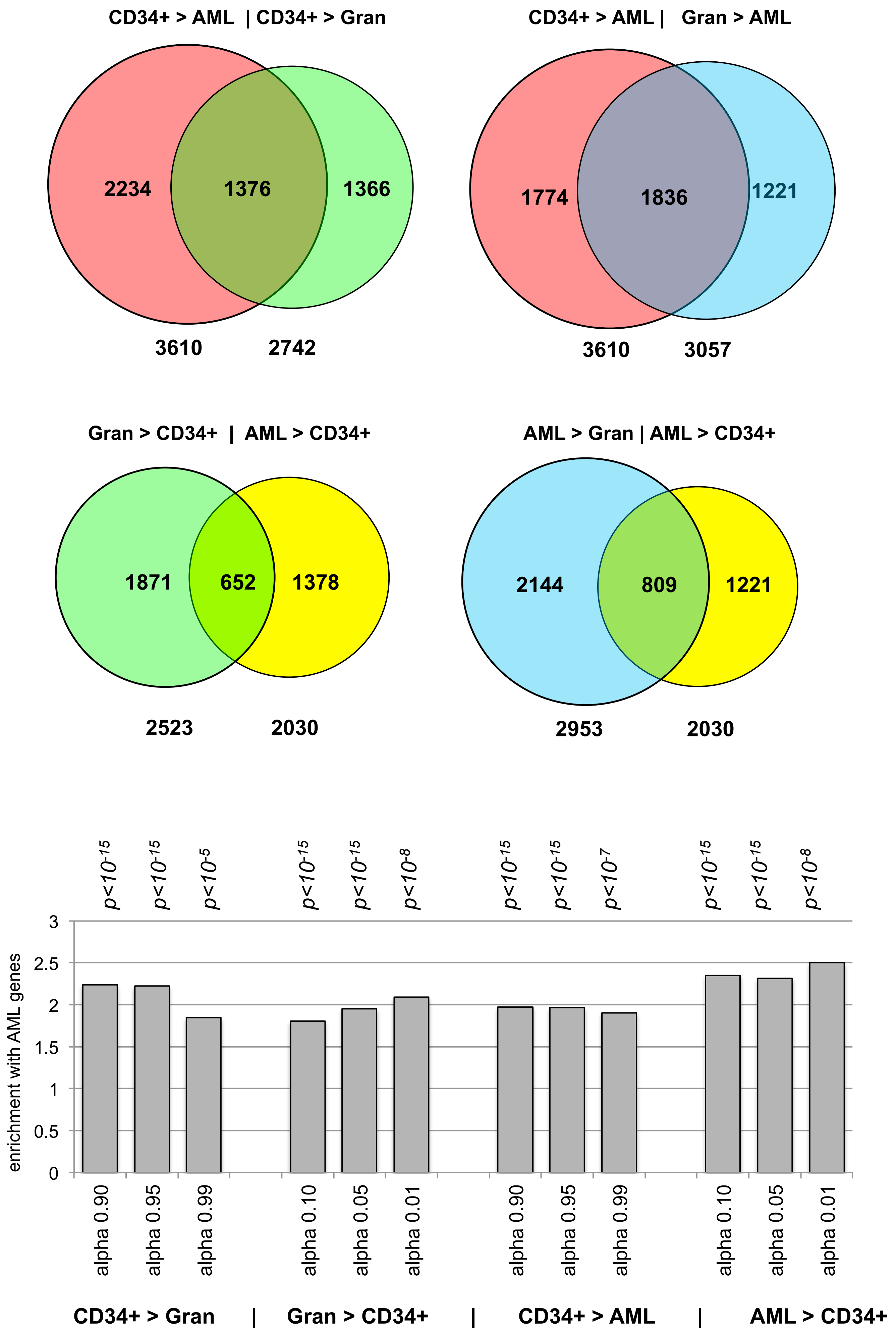

Fig H. H3K9me2 dLOCKs contain both unique and common genes and are enriched with AML-associated genes.

Top: Venn diagrams showing the numbers of unique and common genes within eight types of dLOCK: $dLOCK^{CD34+>AMLA}$, $dLOCK^{CD34+>granulocyte}$, $dLOCK^{granulocyte>AMLA}$, $dLOCK^{AMLA>CD34+}$, $dLOCK^{granulocyte>CD34+}$, and $dLOCK^{AMLA>granulocyte}$ (a>0.95; a<0.05).

Bottom: Graph showing fold enrichment of the four types of dLOCKs: $dLOCK^{CD34+>granulocyte}$, $dLOCK^{granulocyte>CD34+}$, $dLOCK^{CD34+>AMLA}$, and $dLOCK^{AMLA>CD34+}$ (a>0.90,>95, >99 and a<0.10, <0.05, <0.01) with AML-associated genes identified by Ingenuity® pathway analysis (Table B in S7 Tables). Fisher test p-values are shown on top of the graph.

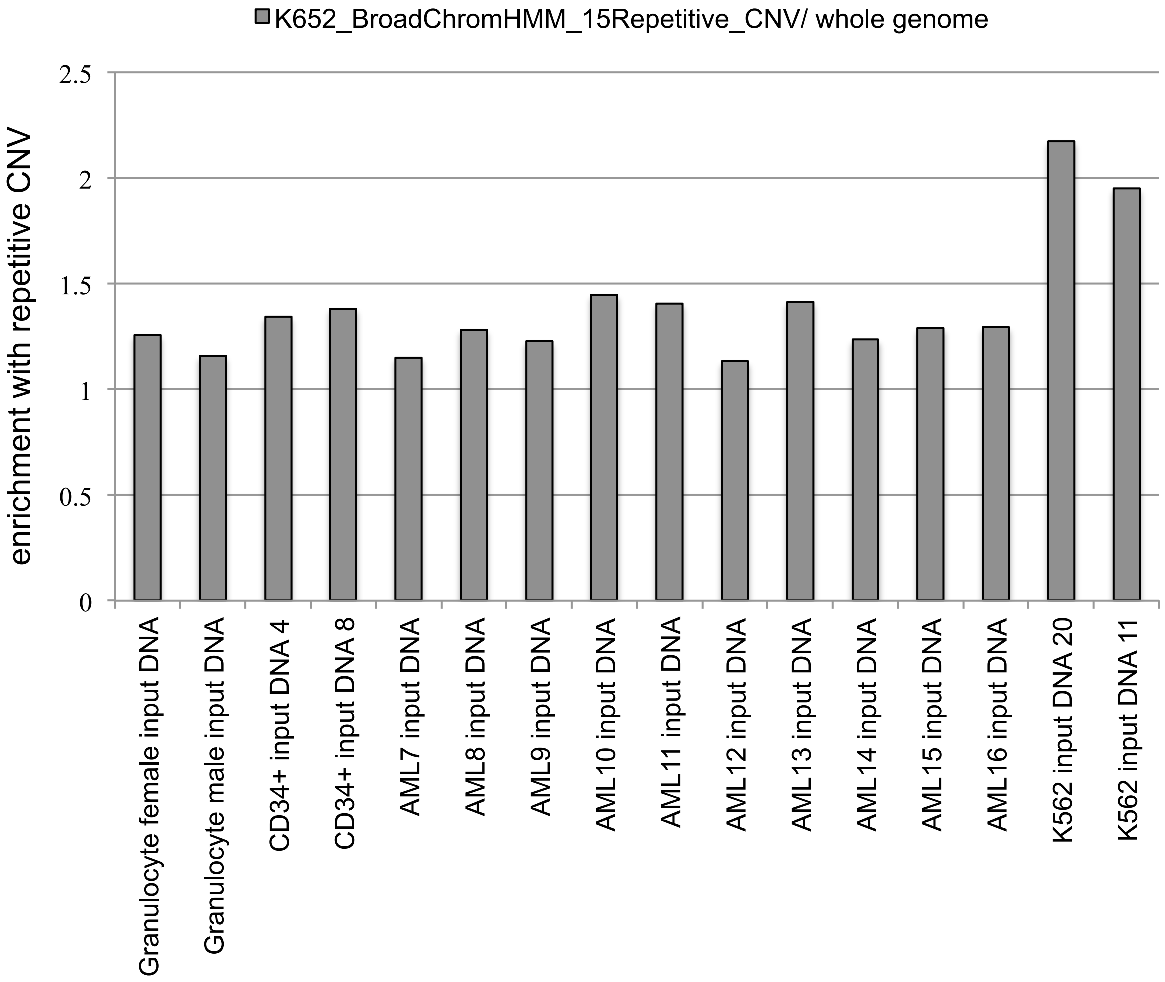

Fig I. DNA copy number variations in myeloid samples.

Bar graph showing genome-wide fold enrichment (over genome average) of the repetitive copy number variations from ENCODE/Broad Institute [43] in the input DNA of 10 AML samples, CD34+, granulocytes, and K562 cells.
