# Supplementary material for: Genome-wide mapping of histone H3K9me2 in acute myeloid leukemia reveals large chromosomal domains associated with massive gene silencing and sites of genome instability
Source: PLoS One. 2017 Mar 16;12(3):e0173723. doi: 10.1371/journal.pone.0173723 (PMC5354391; doi:10.1371/journal.pone.0173723)
Supplement: S8 Table — (DOCX) [file pone.0173723.s009.docx]

S7 TABLE.

| Supplemental S7 Table: PCR primer sequences | | |  |
| --- | --- | --- | --- |
|  | **Gene** | **Forward primer** | **Reverse primer** |
| ChIP-qPCR primers | | |  |
|  | NLRP11 | CGCTCTATACGACACCAGGAG | GAATTGCTCCCTTGCCATTG |
|  | ZNF274_A | TGTCTTGCTCTGCTTTTGACTTAC | CCTGGTTCAGTGTGAGGACC |
|  | ZNF274_B | GAAGATGGAAGCCTGAGTGC | TATAACGGAACTGCCGGAAC |
|  | ZNF544 | ATCTGTGTGCTTCGAGGATGT | GAGACAATATGCTCCCAGGTCT |
|  | TRIM28 | AACATTGCAGAAGAGCACCAA | AGGTCAGGCTAGGTAGGGTCTT |
|  | MECOM | GAACCATCTGAAGCAGGTCTTG | GGCAGTAGGAGTAGAGCCAGTG |
|  | ETS1 | CAAGATCCTTTTAGGCCAAGC | TTCTGGATAGGCTGGGTTGA |
|  | ERG | GTTCTCTCCAGGGCACTCATC | CCAGGTGACAGGCGACAC |
|  | CDH1 | GTGAACCCTCAGCCAATCAG | TCACAGGTGCTTTGCAGTTC |
| RT-pPCR primers | | |  |
|  | ZNF274 | CTGGGTTTTACCCCGGAAG | GCCAGAAATCTTCTGCCTCCT |
|  | ZNF544 | CTGAGGACCTCTGCCCTCTA | GCCATAGCCACATCCTCGAA |
|  | TRIM28 | AACATTGCAGAAGAGCACCAA | AACATTGCAGAAGAGCACCAA |
|  | MECOM | AGTGCCCTGGAGATGAGTTG | TTTGAGGCTATCTGTGAAGTGC |
|  | ETS1 | AGAAGTCGTCACCCCAGACA | GGGTGAGGCGATCACAACTA |
|  | CDH1 | TGGAACAGGGACACTTCTGC | TTCTTGGGTTGGGTCGTTGT |
|  |  |  |  |
|  | ERG | Hs_ERG_1_SG (Qiagen cat# QT00074193) | |
|  |  | *sequence not available* |  |
